# Polyaromatic hydrocarbon inner-structured carbon nanodots for interfacial enhancement of carbon fiber composite†

**DOI:** 10.1039/c9ra08128c

**Published:** 2020-01-02

**Authors:** Xian F. Xi, Yao Y. Li, Liu He

**Affiliations:** Zhongtian Fluorine-Silicone Material Co., Ltd., Zhongtian Group Quzhou Zhejiang 324004 P. R. China; Ningbo Institute of Material Technology and Engineering, Chinese Academy of Sciences Ningbo Zhejiang 315201 P. R. China heliu@nimte.ac.cn

## Abstract

It is well known that carbon substances with a polyaromatic hydrocarbon (PAH) inner structure only form at high temperature. In this work, we introduce fabrication of massive and PAH inner-structured carbon nanodots (CNDs) *via* hydrothermal treatment of glucose aqueous solution in the monolithic methyl silicone hydrogel at 200 °C. During the carbonization process, all the precursor solution is confined in nano-vessels (2–20 nm) of the thermostable methyl silicone hydrogel, thus forming CNDs without aggregation. The resulting CNDs, with a yield of 65%, were separated facilely and characterized using various spectroscopy and microscopy techniques. The glucose-derived CNDs have diameters of 2–5 nm and contain 18.9 wt% carboxyl groups, and their aqueous solubility depends on the pH. The CNDs consist of large PAH clusters, confirmed by solid-state ^13^C NMR, which were different to other reported carbon substances prepared at similar low temperatures. The formation mechanism of the PAH structure in the CNDs probably relates to the high interfacial energy of the prewetted superhydrophobic methyl silicone nano-framework in the hydrogel. Moreover, the tunable fluorescence properties of the CNDs prepared using this method can be attributed to the arene carboxylic groups in the CNDs. Finally, the resultant PAH CNDs with abundant groups were applied as a sizing in carbon fiber (CF) composite fabrication, resulting in an obvious interface enhancement of the CF/epoxy composite.

## Abstract 2

Aggregation-free fabrication of polyaromatic hydrocarbon inner-structured carbon nanodots *via* hydrothermal treatment of the glucose aqueous solution in the monolithic methyl silicone hydrogel is introduced.

## Graphical abstract

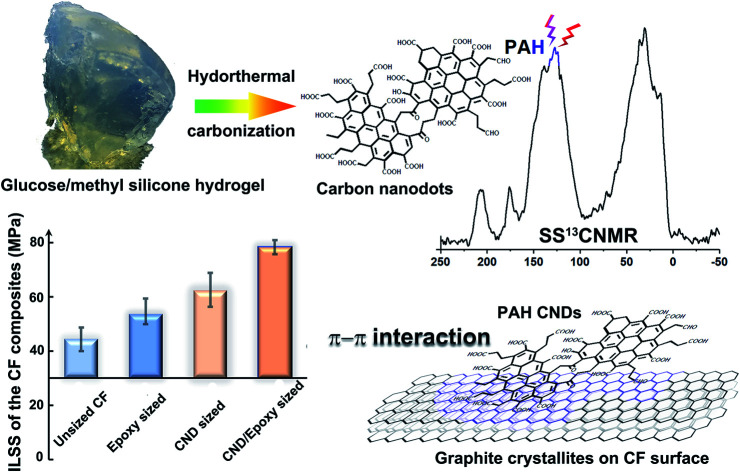

## Introduction

Carbon nanodots (CNDs) are found in the fragments of multiwalled carbon nanotubes and are increasingly being explored because of their wide range of applications in ion and organic molecule detection, temperature sensing, optoelectronic and energy technologies, photocatalysis, corrosion inhibition, and bio-imaging.^1,2^ Due to the flexible design of the composition of CNDs, great efforts have also been devoted to developing methods for the preparation of CNDs from small molecule precursors by a bottom-up route.^3,4^ However, for most bottom-up methods, such as hydrothermal, solvothermal, microwave, fused salt and so on, there are still difficulties in controlling the aggregation of CNDs, because the strong surface tension of these nano-sized particles leads to random agglomerations during the carbonization process.^1^ These agglomerations lead to large-sized, insoluble byproducts. Especially in hydrothermal preparations, high yield of CNDs with a deep carbonized structure only has been reported rarely because the CNDs form in hydrothermal solutions with uncontrolled precipitate byproducts.^5,6^ To prevent aggregation during preparation, the temperature was reduced, or the reaction time shortened, compared with conditions used in the preparation of hydrothermal carbon (HTC).^7^ Salts or surfactants as a soft template have also been added to stabilize the CNDs in the hydrothermal solution.^8,9^ Silica microspheres have been reported to serve as a hard template and nucleating agent in the preparation of CNDs.^10,11^ Porous silica powders with nano-size pores are used as templates for preparation of CNDs with a narrow particle size distribution.^12^ Only a portion of the precursors is absorbed in the limited internal porosity of the template to form CNDs, and the precipitates form outside the micrometer-sized template.

Large numbers of reports focus on the applications of bottom-up CNDs, whereas it might be better to pay more attention to the inner chemical structures of the CNDs. Based on the degree of carbonization of the carbon core, the carbon dots are classified into graphene quantum dots, carbon nanodots and polymer dots.^13^ For bottom-up CNDs prepared from molecules below 300 °C, the carbon cores consist of amorphous or polymer structures.^14,15^ In some sense, these bottom-up CNDs are just polymer dots.^16^ The microwave-synthesized CNDs were confirmed to have a supramolecular structure by ^13^C NMR.^6^ Hydrothermal cellulose carbon dots have also been certified as having polymer furan inner structures by ^13^C NMR, even in hydrothermal samples treated at 260 °C.^17^ This polymer carbon structure is in accord with classical hydrothermal carbonization research, whereby the hydrothermal carbons have a polymeric carbon structure with an obvious furan feature, even after lengthy hydrothermal treatment at 280 °C.^18^ Deep carbonization of hydrothermal CNDs at low temperature is worth exploring.^19^ The π–π stacking interaction of aromatic dimers has been widely reported in research on the mechanism of CND fluorescence,^1,2,16^ as well as in research on crystal engineering, aromatic polymer self-assembly, and supramolecular interactions.^20,21^ The π–π interactions are responsible for the strong adsorption of organic molecules onto graphene materials, especially aromatic molecules. However, there are very few reports on CNDs applied to the fabrication of carbon fiber (CF) composites, which have reached a mature stage of development as an engineering material for high-performance applications, owing to their highly specific mechanical properties. CF is a graphite crystallite substance with a skin–core structure. Graphite crystallites are of larger size and higher density at the top surface of the CFs.^22^ Due to the low polar graphite crystallite-rich skin of the CFs, the smooth and inert surface of the carbon fiber usually results in poor wettability and interfacial adhesion with the resin matrix.^23^ To improve the performance of CF composites, methods for modification of the CFs have been developed, such as surface treatment of carbon fibers, including fiber sizing and coating, electrochemical oxidation, liquid-phase chemical oxidation, high energy beam irradiation plasma treatment, *etc.*^24^ Nanofiller materials, including carbon nanotubes, inorganic nanoparticles, graphene and ceramic whiskers, have been incorporated into sizing materials to form an enhanced interphase and to contribute positively to the interfacial bonding of carbon fiber-reinforced composites. However, direct interaction between the nanofillers and CFs is rarely reported. The large polyaromatic hydrocarbon (PAH) inner structures of deep carbonized CNDs probably have a strong π–π interaction with the graphite substance and, considering the abundant polar groups of the CNDs, the CNDs are anticipated to enhance the interfacial adhesion of the carbon fibers/resin composite.

**Fig. 1:**
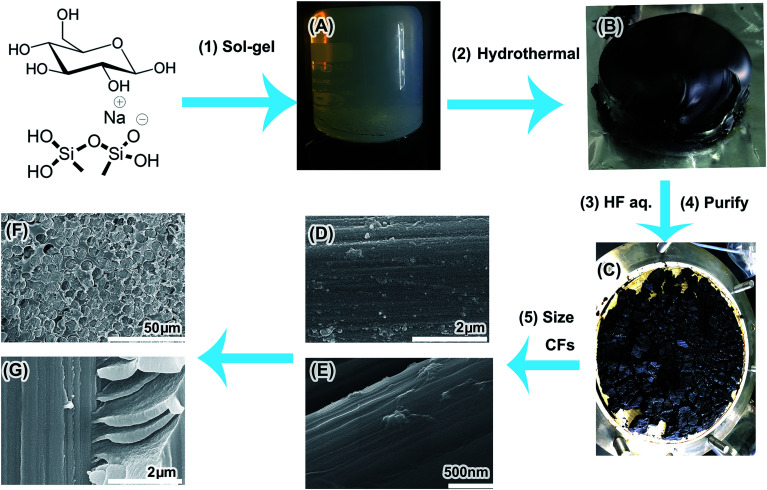
Preparation of the PAH CNDs and their application in CF composites. (A) MGSH, (B) carbonized MGSH, (C) purified CNDs, (D) SEM image of CFs sized by CNDs, (E) SEM image of CFs sized by CNDs/epoxy sizing, (F and G) SEM images of the CF/epoxy composites enhanced by CNDs.

Herein, based on the merits of earlier research, a facile method to fabricate a high yield of PAH CNDs without insoluble byproducts *via* hydrothermal treatment of the monolithic glucose/methyl silicone hydrogel (MGSH) is introduced. The MGSH was prepared facilely from glucose solution, HCl solution and sodium methyl silicate solution by the sol–gel method (Fig. 1). The resultant CNDs were purified simply and the large PAH inner structures confirmed by solid-state ^13^C NMR. A formation mechanism for the deep carbonized structure of the CNDs is proposed. The CNDs were applied in the sizing of carbon fibers, clearly improving the interfacial adhesion of the CF/epoxy composite.

## Experimental section

The sodium methyl silicate solution (solid content 42 wt%, Na_2_O content 10 wt%) and sodium silicate solution (solid content 42 wt%, Na_2_O content 10 wt%) were supplied by Zhejiang Zhongtian Fluorine-Silicone Material Co., Ltd, Zhejiang, China. The T700 CF textile was supplied by ZhongFu ShenYing Co., China. Epoxy sizing emulsion (40 wt%, CFC-AS-K7) was supplied by Shanghai Organic Chemistry Research Institute, CAS. Epoxy resin E-44 and Polyamide Curing Agent 650 were purchased from Nantong Xingchen Synthetic Material Co. Ltd., Nantong, China. Other chemicals were purchased from Sinopharm Chemical Reagent Company and used as received without further purification. Deionized water (18.2 MΩ cm at 25 °C) was used in all experiments.

### Preparation of MGSH

The preparation procedure for the MGSH CNDs is shown in Fig. S1.† First, 250 g 40 wt% glucose solution was prepared by dissolving 100 g of d-glucose monohydrate in 150 g of deionized water. Next, 72 g of HCl solution (18 wt%, 36.5 wt% HCl diluted with ultra-pure water) was blended with the glucose solution, and then the HCl–glucose solution and 180 g of sodium methyl silicate solution were poured into a 500 ml beaker with vigorous stirring for 30 seconds. Finally, the translucent MGSH formed, with a glucose content of 18.3 wt%. The appearance and porosity of the MGSH were dependent on the quantity of HCl added. In each sample, 10 g MGSH was cut and soaked in 3000 ml deionized water for 48 h and freeze-dried for Brunauer–Emmett–Teller (BET) characterization. All the CNDs discussed in this paper were made from MSGH prepared using the above procedure, unless stated otherwise. This MGSH was called sample 1. For comparison, the monolithic silicone hydrogel with 10 wt% glucose and 10 wt% ethanediamine is called sample 2.

### Preparation of CNDs by hydrothermal treatment of MGSH

The MGSHs and N-hybrid MGSHs were sealed in beakers and then sealed in a 2000 ml Teflon-lined autoclave with 50 ml water, which maintained the vapor pressure balance. After heating at 200 °C for 24 h, the translucent MGSHs transformed into black CND silicone hydrogels or brown N-doped CND silicone hydrogels. For the hydrothermal-treated MGSHs, the CND hydrogel was broken into a brown slurry, and the pH was adjusted to 1 with HCl solution. The slurry was washed and filtered five times with abundant deionized water, and the filter residue was soaked in 500 g of 40 wt% HF solution in a polypropylene flask. (**Caution**!! Add HF solution into the filter residue slowly in a fume cupboard! Keep from boiling!) After reaction and vaporization for 48 h, all of the silicone framework turned into CH_3_SiF_3_ gas and was removed, and the remaining CNDs were filtered and washed five times using 20 times weight of deionized water. The weight of the wet CNDs varied between 138 and 145 g, with 75 wt% moisture content. A portion of the wet CNDs was dried at room temperature in a vacuum oven for 24 h, and then sealed in polypropylene (PP) valve bags for the tests. The yield was 65% (calculated by carbon atoms). Only the CND solutions were prepared from the wet CNDs. The first-wash water was collected for fluorescence characterization, which is shown in Fig. S1A.† The hydrazine hydrate-modified MGSH CNDs were prepared by stirring hydrazine hydrate (50 g, 50 wt%) and as-synthesized MGSH CND ethanol solution (50 g, 0.5 wt%) at 90 °C for 24 h (see Fig. 3D for the fluorescence emission and excitation). After hydrothermal carbonization of the N-hybrid MGSH, sample 2 was first extracted using deionized water and then extracted using DMF. The N-hybrid MGSH CNDs have excellent solubility and can be extracted completely from the silicone frameworks by solvents (Fig. S2†). The hydrothermal-treated MGSH slurry was treated with insufficient HF solution and washed according to the above method. The remaining CNDs were dried at room temperature in a vacuum oven for 24 h and are denoted as CNDs@silicone. In the contrasting experiments with glucose/silica hydrogel hydrothermal carbonization, the sodium silicate solution was used instead of sodium methyl silicate solution.

**Fig. 2:**
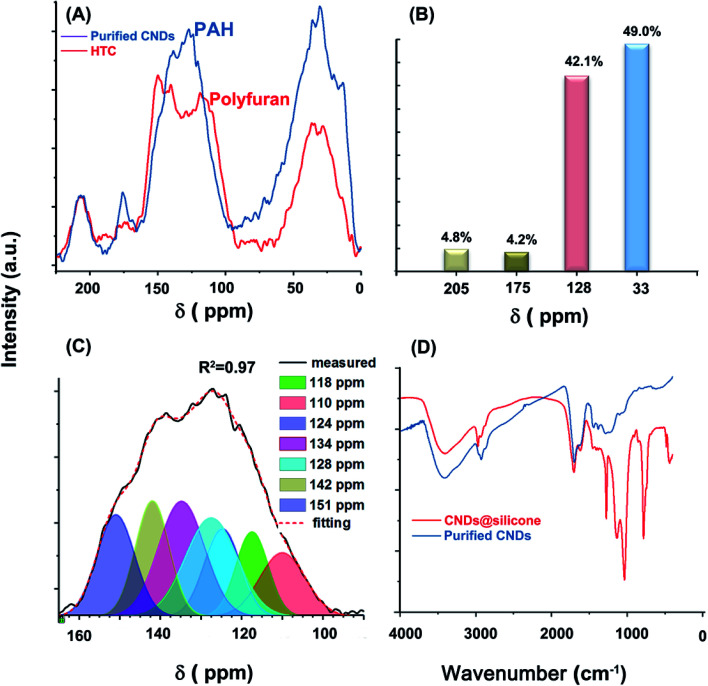
(A) ^13^C CP MAS TOSS NMR spectra of MGSH CNDs and that of HTC spheres prepared under the same conditions. (B) Integration of four peaks of the CND spectra. (C) Curve-fitting for 90–165 ppm of the CND NMR spectrum. (D) Contrasting the FT-IR spectra of MGSH CNDs and that of CNDs with silicone.

**Fig. 3:**
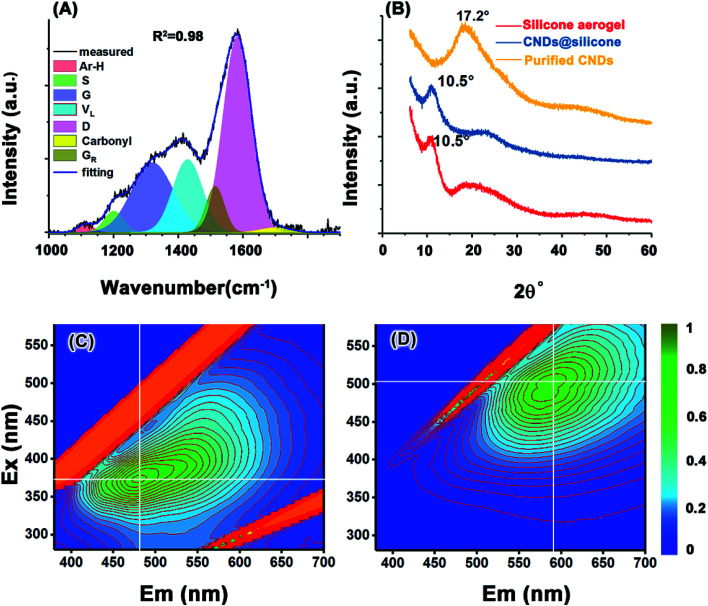
(A) FT Raman of the MGSH CNDs, (B) XRD spectra of the MGSH CNDs. Excitation–emission matrix for (C) the MGSH CNDs and (D) the hydrazine-reduced MGSH CNDs.

### Preparation of hydrothermal carbon spheres

The preparation was carried out according to the reported method.^18^ Aqueous glucose solution (600 g) with a content of 10 wt% was sealed in a glass tube, and then sealed in a 2000 ml Teflon-lined autoclave with 50 ml of water, which maintained the vapor pressure balance. After heating at 200 °C for 24 h, brown-black sediments formed. After washing and filtration with deionized water, about 24 g of remaining sediment was dried in a vacuum oven at 120 °C for 24 h before analysis with solid-state ^13^C NMR spectroscopy.

### Fabrication of unidirectional CF/epoxy composite short beams

The as-received CF textiles were extracted in acetone by Soxhlet extraction for 48 h to remove the epoxy sizing and pollutants. Then, the extracted textiles were washed with deionized water until acetone was completely removed, and finally dried in a vacuum oven at 50 °C. Then, the unsized carbon fiber textiles were saved in a PP valve bag. The CF bundles were pulled out from the unsized CF textile and dipped into the 0.1 wt% CND solution (CNDs were dissolved in 50 wt% ethanol aqueous solution), 1.0 wt% epoxy emulsion (the as-received sample was diluted with deionized water) and the epoxy/CND sizing, respectively. The epoxy/CND sizing was prepared by blending 90 g of 1.0 wt% epoxy emulsion and 10 g of 0.1 wt% CND solution. After drying, every 15 CF bundles (12 000 fibers per bundle) were placed in a mold unidirectionally, immersed with epoxy (67 wt% E44 resin and 33 wt% Polyamide Curing Agent 650) and bubbles removed in vacuum. After excessive epoxy was pressed out by a press-holding vulcanizer, the samples were cured at 80 °C for 24 hours. The samples were 2 ± 0.2 mm × 6 ± 0.1 mm ×250 ± 5 mm, with 55 ± 1 wt% CF content, as shown in Fig. S3.† The CF bundles were sized by 0.01 wt% CND solution only for CF topographic comparison.

### Characterizations

The specific surface area and pore size of the MGSHs were determined by physical adsorption using a Micrometrics ASAP 2020 HD88 physisorption analyzer. Fourier transform infrared (FT-IR) spectra were obtained using a Nicolet 6700 FT-IR spectrometer, Thermo Scientific Co., Ltd, USA. The liquid-state ^13^C NMR spectra were recorded on a Bruker AVANCE III 400 spectrometer. The solid-state cross-polarization (CP)/magic-angle spinning (MAS)/total sideband suppression (TOSS) ^13^C NMR spectra were recorded on a Bruker AVANCE III 400 WB spectrometer. Carbon, hydrogen, oxygen, nitrogen (CHON) element analysis was performed on an Elementar EL Cube. Transmission electron microscope (TEM) analysis of the samples was performed using a Tecnai F20 microscope. Raman spectroscopy analysis was performed on a Renishaw inVia Reflex confocal Raman microscope at 542 nm excitation wavelength. Before testing, the CND sample was heat-treated at 160 °C for 12 h in a vacuum oven to eliminate fluorescence. The data treatment was performed using Origin Pro 2017 software. X-ray photoelectron spectroscopy (XPS) analysis was performed using an AXIS ULTRA X-ray photoelectron spectrometer with a MgK X-ray source (1253.6 eV) operated at 14 kV and 300 W, with an emission current of 25 mA. Survey scans were collected from 0 to 1100 eV. The samples were examined using scanning electron microscopy (SEM) on a Hitachi 4800 and Hitachi TM 1000 microscope at accelerating voltages of 5–8 kV and 15 kV, respectively. The carboxyl group contents were confirmed by pH titration of the CNDs. Photoluminescence spectra were obtained using a Hitachi F-4600 spectrophotometer at ambient conditions. Three-point short-beam shear tests were performed on an Instron mechanical test machine (Instron 5985), according to the ASTM D2344M standard.

## Results and discussion

**Fig. 4:**
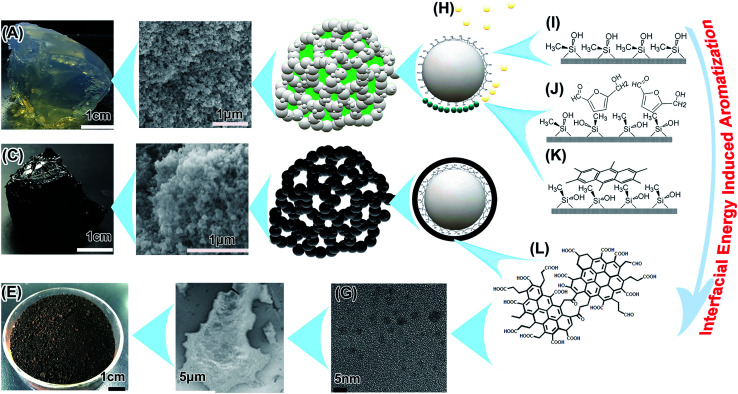
Supposed interfacial energy inducing aromatization. (A) The MGSH with glucose content of 18.3 wt%. (B) SEM image of the MGSH framework after freeze-drying. (C) MGSH after hydrothermal treatment. (D) SEM image of hydrothermal-treated MGSH after freeze-drying. (E) Dried CNDs. (F) SEM image of CNDs. (G) TEM of the CNDs. (H) Hydrothermal intermediates. (I) Unstable distorted conformation of the superhydrophobic silicone framework surface in the solution. (J) Adsorption of the hydrothermal intermediates at the high energy interface. (K) Carbonization of the two-dimensional adsorption layer. (L) The supposed CND inner structure.

After the hydrothermal process, MGSH keeps its shape and inner structure well (Fig. S4†). The methyl silicone framework of the MGSH was decomposed and vaporized as CH_3_SiF_3_ gas, by treatment with hydrofluoric acid (HF) solution. The residues were washed with water and dried, with a yield of 65% (calculated by carbon atoms). The resultant brown powder was confirmed as CNDs with a deep carbonized structure by FT-IR spectroscopy, ^13^C CP TOSS MAS NMR, FT Raman spectroscopy, HRTEM, XPS and CHON element analysis. The fluorescence properties of the CNDs and their reduced product were also investigated and compared by fluorophotometry. The MGSH has a porous methyl silicone framework, with a high BET surface area (537.33 m^2^ g^−1^) and a pore diameter range of 2–20 nm (Fig. S5†). During the hydrothermal carbonization, all the glucose solution was contained within the nano-vessels of the MGSH, and the methyl silicone framework of the MGSH limited the range of CND movement to avoid CND collisions, thus preventing the formation of large byproduct particles.^9^ The silicone framework also served as nucleation centers,^25^ and carbon grew at the interface during the hydrothermal carbonization, owing to the high interfacial tension of the silicone and aqueous solution confined in the hydrogel. The resultant CNDs can dissolve completely in water, alcohol, acetone and tetrahydrofuran, indicating that no large-sized hydrothermal carbon (HTC) particles form in the MGSH, even after lengthy hydrothermal treatment at 200 °C. The diameter of the CNDs ranged from 2 to 5 nm, as obtained from the TEM image (see Fig. 4G). The water solubility of the CNDs varied from 200 to 5000 ppm depending on the solution pH (Fig. S6†). The surface functional groups of the CNDs are mainly carboxyl groups (18.9 wt%, Fig. S7†). There was no signal in the liquid-state ^13^C NMR spectrum for the CND methanol-d solution (Fig. S8†), suggesting that the CNDs are condensed colloids in the solution, not soluble molecules. But the N-hybrid CNDs have excellent solubility and were certified as a thick liquid after removal of solvent.

### PAH inner structure of the CNDs

The glucose-derived CNDs have a typical aromatic carbon and aliphatic carbon structure, more similar to that of brown coal or pyrolytic carbon at high temperature, and different to the poly-furan structure widely reported in HTCs. As shown in Fig. 2A, the ^13^C CP MAS TOSS NMR spectrum of the MGSH CNDs contains four peaks near 205, 175, 128, and 33 ppm, corresponding to keto C, carboxyl C, aromatic C, and aliphatic C, respectively. The aromatic C peak differs from that of the classical furan-rich carbon structure reported in HTCs and hydrothermal CNDs (Fig. 2A and S9†).^26,27^ The Gaussian curve-fitting of the aromatic sp^2^ peak is shown in Fig. 2C. For HTC, the peaks at 110 and 151 ppm correspond to the furan structure, and the peaks at 118–141 ppm correspond to the arene structure.^18^ The ratio of the furan structure sp^2^ C to the arene C is an indicator of the degree of carbonization. In this work, the peak area ratio of furan sp^2^ C (110 ppm) to aromatic C (128 and 134 ppm) is far below that of HTC prepared under similar conditions, and corresponds to a deeper degree of carbonization.^28^ The aromatic C with a high content near 128 and 134 ppm belongs to bridge C atoms, which indicates the PAH inner structures.^29,30^ The obvious peak at 142 ppm indicates that the methyl or methylene groups link directly with the aromatic C.^31^ Comparison of the solid-state ^13^C NMR spectra of MGSH CNDs to those of graphene oxide and reduced graphene oxide also indicates PAH inner structures in MGSH CNDs (Fig. S10†). The large area under the peak at 42.5 ppm indicates a high content of quaternary or naphthenic C atoms (Fig. S17†).^30,32^ The CHON ultimate analysis of the CNDs and classical HTC are listed in Table 1. The H element content determined for the CNDs is 30% higher than that of classical HTC,^33^ which is in accord with the more aliphatic C atoms determined by ^13^C NMR (Fig. 2B).

**Table 1:** CHON element analysis of the CNDs

Element^a^ (wt%)	C	H	O	N	Total
CNDs	65.62	6.02	27.77	0.38	99.79
CNDs@silicone^b^	63.12	5.67	28.67	0.08	96.78
HTC	64.47	4.69	30.85	—	100.1^c^

a Analysis error less than 0.2%.

b CNDs with silicone were the CNDs treated with insufficient HF solution at purification.

c Classical HTC data reproduced with permission,^33^ copyright Year 2008, Royal Society of Chemistry.

The amount of aromatic H atoms (mole) in 100 g of CNDs was calculated using Fig. 2B and Table 1 assuming a CH_2_/CH_3_ ratio of *R*:

1 Aromatic H = total H − carboxyl H − nitrogen H − aliphatic H

Substituting in the values based on Fig. 2B, Table 1 and eqn (2) is established.

2 Aromatic H = 0.41–2.68/(*R* + 1)

As shown in eqn (2), it can be inferred that the aromatic H atoms in 100 g of CNDs approaches 0 mole as *R* approaches 5.53. Therefore, *R* must be over 5.53, and there must be few methyl groups in the CNDs. This finding is consistent with the 18.9 wt% content of carboxyl groups in the CNDs, which replace the methyl groups at the ends of the aliphatic chains. Considering the obvious methyl shoulder peak at 2960 cm^−1^ in the FT-IR spectrum (Fig. S15C†), *R* should approach 5.53 and the aromatic H content should approach zero. This agrees with the absence of an obvious peak for aromatic H bending near 800 cm^−1^ in the FT-IR spectrum (Fig. 2D). The aromatic H content of the CNDs (chemical shift 90–125 ppm) is obviously lower than that of lignites and is comparable to inertinite-rich coal, according to comparison of the sp^2^ peaks in the solid-state ^13^C NMR (Fig. S11†). The lower aromatic H content indicates a larger conjugated PAH structure, but perhaps not larger than an eight-ring PAH, as calculated using the classical method reported (Table S3†).^34–36^

The CNDs have obvious IR absorption peaks of OH (3400 cm^−1^), CH_2_ (2927 cm^−1^), C=C (1620 cm^−1^), and C=O (1701 cm^−1^), indicating the presence of aromatic structures and methyl, methylene, and O-containing groups (Fig. 2D). In contrast to previously reported glucose-based hydrothermal CNDs and HTC spheres,^8,15,37^ very few characteristics of the glucose remained in the CND fingerprint IR region at 400–1500 cm^−1^. The typical aromatic C–H bending vibration near 800 cm^−1^ is not obvious, in comparison with that of hydrothermal carbon spheres prepared under the same conditions.^37^ In addition, the characteristic peak of long aliphatic alkanes ((CH_2_)_*n*_; *n* > 4) near 680–720 cm^−1^ was not observed. Unlike purified CNDs, the CNDs with silicone (blue line) have very strong absorption peaks at 2960, 1272, 1132, 1031, and 783 cm^−1^, which belong to the CH_3_ of silicone and types of Si–O groups.^12^ Curve-fitting of the CND FT-IR spectra was performed to obtain further details, as shown in Fig. S15A–C and Table S2.† Overall, the fine shape of the FT-IR spectrum accords with that of some graphene oxides, some graphene quantum dots and some brown coals (Fig. S13†) in the 1000–4000 cm^−1^ region, and especially in the 1000–1500 cm^−1^ region.^38–41^

The Raman spectrum of the heat-treated CNDs (Fig. 3A) contains a broad peak near 1360 cm^−1^ and a sharp peak at 1587 cm^−1^, which does not directly represent the defects and graphite structure for a hydrothermal immature carbon.^42,43^ The CH_*x*_ stretching band occurs at 2930 cm^−1^ (Fig. S12A†).^44^ Gaussian curve-fitting shows very weak aromatic H and carbonyl bands at 1113 cm^−1^ and 1700 cm^−1^, respectively.^45,46^ The band at 1200 cm^−1^ belongs to the S band, indicating an aliphatic ring. The V_L_ band appears at 1428 cm^−1^, which is associated with methylene or methyl and the semicircle breathing of aromatic rings. The G_R_ band at 1513 cm^−1^ and G band at 1583 cm^−1^ correspond to the semicircle breathing of the aromatic rings and the in-plane bond-stretching motion of pairs of carbon sp^2^ atoms, respectively. The D band at 1320 cm^−1^ corresponds to the breathing mode of A_1g_ symmetry.^47,48^ The D/G intensity ratio is 0.52 (Table S1†), given the D position of 1320 cm^−1^ and the G position of 1583 cm^−1^. It can be inferred that the ring number of the PAH cluster in the CNDs is approximately 8–12, based on the standard curve reported.^42^ A broad band appears at 17° in the X-ray diffraction (XRD) spectrum of the MGSH CNDs (Fig. 3B), corresponding to a d-spacing of 0.52 nm. Because silicone impurities enlarge the d-spacing, the obvious peak of the CNDs with silicone appears at 10.5°, which corresponds to the XRD pattern of the methyl silicone aerogel framework (red line, Fig. 3B) and previous reports.^49,50^ The XRD main peaks of previously reported CNDs usually vary from 17° to 26°, indicating the different distances between the adjacent carbon atomic planes of the CNDs.^15,51–54^ These XRD results do not indicate a graphite or graphene structure carbon atomic plane directly, because of similar results in polymer research. The ^13^C NMR, FT Raman spectroscopy, and FT-IR spectroscopy results confirm that the MGSH CNDs in this work have PAH carbon atomic planes.

Fluorescence is one of the important characteristics used in CND research and is essentially determined by the structure of the CND. For sucrose CNDs, mini-sized blue fluorescence CNDs and bigger green fluorescence CNDs can be separated using a dialysis bag (*M*_w_ 3500).^8,55^ A similar phenomenon was found in the CND purification process. After washing with water for the first time, bright blue fluorescence was observed in the waste water (Fig. S1A†), and the remaining purified CNDs exhibited green fluorescence (Fig. S1B†). Fig. 3C and D presents the optical characterization results for the MGSH CNDs and hydrazine-reduced MGSH CNDs. For the MGSH CNDs, the maximum emissions gradually shift from 450 to 550 nm when the excitation wavelength changes from 350 to 390 nm (Fig. 3C). For the hydrazine-reduced MGSH CNDs, the maximum emissions range from 560 to 630 nm, with the excitation wavelength changing from 480 to 520 nm (Fig. 3D), indicating that hydrazine-reduced MGSH CNDs lead to longer wavelength emission, which is different from graphene oxide quantum dots and leads to shorter wavelength emission after reduction with hydrazine hydrate.^56^ For the bottom-up carbon dots, it is well known that the longwave emission feather is in accordance with the reported arene amine-based carbon dots. It may be inferred that the carboxyl groups on the arene structure of the CNDs may be turned into arene amine structures in the hydrazine reduction.^4,57^ Therefore, the arene carboxyl groups exist in the CNDs, which is in accordance with the curve-fitting results of the FT-IR and Raman spectra (Fig. S12, S15, Tables S1 and S2†).

### Supposition of PAH structure formation under mild conditions

It is still a mystery that a deep carbonized structure forms under such mild hydrothermal conditions. Perhaps a different carbonization mechanism at the interfacial tension is inducing this condition (Fig. 4), and this needs further confirmation. There were some special hydrothermal carbonization conditions in this study. First, limited nano-vessels confine the movements of the precursors and intermediates, and this is adequately discussed elsewhere.^9,10,12,58,59^ Second, the huge surface areas of the templates induces interfacial hydrothermal carbonization. There are vast numbers of reports on 2–50 nm-thick carbon-rich polysaccharide pretreatment coating on the surface of dispersed nanoparticles and colloids by hydrothermal carbonization, pyrolyzed for electroconductivity improvement of energy storage materials.^60–64^

But in the MGSH, the high interfacial energy^65^ may benefit the aromatization. The framework of MGSH, is a superhydrophobic substance (just a methyl silicone aerogel after freeze-drying, Fig. S4†), wetted completely by the glucose solution with high interfacial energy in the MGSH formation process.^66–68^ To reduce the high interfacial energy, the methyl silicone framework keeps an unstable distorted conformation in solution (Fig. 4G). In this hydrothermal treatment, most of the glucose and intermediates lose part of their hydrophilic groups in the complex chemical process, decrease in water solubility and form amphiphilic substances (partly soluble substances).^69–72^ They then adsorb more intensively on the superhydrophobic and oleophilic surface^73,74^ of the silicone nano-framework, and self-assemble^75^ to reduce the high interfacial energy (Fig. 4H). The BET surface of the silicone framework is 537.33 m^2^ g^−1^, and the weight contents of silicone, glucose and CNDs in the MGSH are about 18.0 wt%, 18.3 wt% and 7.0 wt%, respectively. Hence, it can be inferred that 1 g of glucose corresponds to about 500 m^2^ of silicone framework interface, and 1 g of CNDs corresponds to 1400 m^2^. When the limited quantity of glucose intermediates is exposed to the high energy interface with a huge surface area, the intermediates must adsorb intensively at the interface and self-assemble into an orderly two-dimensional adsorption layer,^76,77^ which may promote formation of the planar PAH structure. This would be different to classical hydrothermal carbonization, where polymerization and carbonization take place in a two-dimensional ordered adsorption layer at the silicone interface, not in the intermediate mini-drops or colloids in the solution, in which the intermediate forms a three-dimensional cross-linked polymer structure first.^18^ The three-dimensional polymer structure probably inhibits further aromatization under mild conditions. Therefore, it can be concluded that the high interfacial energy of the methyl silicone superhydrophobic framework in the solution is an important factor in glucose aromatization under such mild hydrothermal treatment.

Amphiphilic intermediates and their aggregations at the high energy interface may play an important role in the aromatization. When ethanediamine is added into the glucose solution in the preparation of MGSH, the carbonization is inhibited in the subsequent hydrothermal treatment. As shown in Fig. S2,† all the N-hybrid CNDs were washed out from the silicone framework by dimethylformamide (DMF). This phenomenon indicates two results. First, the resultant N-hybrid hydrothermal product has a very high solubility in DMF. Second, there are no carbon particles anchored on the silicone framework. After removing the DMF by rotary evaporation, the N-hybrid hydrothermal product turns to a brown thick liquid, which is in accord with the reported polyimide results.^6,78^

Hydrothermal treatment of the glucose/silica hydrogel also leads to aromatization under the same conditions. When the hydrophilic silica framework is used instead of the methyl silicone framework discussed above, the resultant CNDs have an obvious arene inner structure with a peak at 113 ppm in the ^13^C NMR spectrum (Fig. S16†), which is different from the 128 ppm peak recorded for MGSH CNDs by ^13^C NMR. According to research into curve-fitting for ^13^C NMR,^34^ the silica hydrogel CNDs have an obviously smaller arene size than the MGSH CNDs. Perhaps, the stronger interfacial energy results in deeper glucose aromatization. Whether the silica/silicone hydroxyl has catalyzed glucose aromatization requires further exploration.

### Interfacial enhancement of carbon fiber composites by CND sizing

**Fig. 5:**
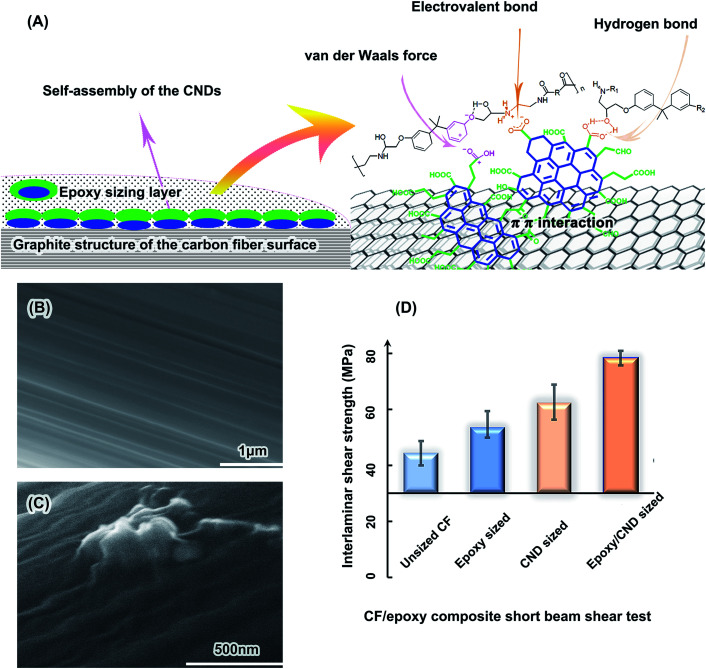
Interfacial adhesion of the CF/epoxy composites improved by CND sizing. (A) Possible enhancement mechanism. (B) SEM image of unsized CF. (C) SEM image of epoxy/CND-sized CF. (D) Interlaminar shear strength of the CF/epoxy composite short beams.

As discussed above, the CNDs have an oleophilic PAH-structured core and abundant polar functional groups, including 18.9 wt% carboxyl groups. Considering the CNDs formed at the interface of the superhydrophobic silicone framework and water solution, it can be inferred that the CNDs have an oleophilic side near to the silicone and a hydrophilic side near to the water solution. This amphiphilic structure results in self-assembly and aggregation at high concentration. The wet-storage CNDs can be dissolved in various polar solvents and the epoxy emulsion. The CND/epoxy emulsion is pale yellow in sunlight and white in 365 nm UV light (Fig. S18†). This uniform appearance and fluorescence of the CND/epoxy emulsion suggests that the CNDs were completely dissolved in the emulsion at low concentration. They can be facilely used in the CF sizing process. On the other hand, the CNDs have limited solubilities (no more than 1% usually) in solvents. When the saturated CND solution is exposed to air, the concentration of the CNDs will increase and become slowly supersaturated, resulting in the CNDs self-assembling and forming a transparent blue film on the surface of the CND solution, to decrease the surface tension of the liquid. In the CF sizing process, CND solution or CND/epoxy emulsion wetted the CFs and became supersaturated in the drying process. Aggregations formed on the surface of the CFs, owing to the nucleation effect of the CFs in the CND supersaturated solution.^79^ The CND aggregations on the CFs increase the roughness of the CF surface, which may increase interlocking between the CFs and the matrix.^80^ For CND solution sizing, the size distribution of the CND aggregations on the CND-sized CF surface varies from 30 to 1000 nm, and is determined by the CND concentration of the sizing solution, as shown in Fig. S19.† A concentration of 0.01 wt% is enough for full coverage of the CFs by CND aggregations. The higher CND concentration leads to larger aggregations. After the epoxy/CND sizing, there is a wrinkled continuous epoxy sizing layer covering the rough CND layer on the CFs, as shown in Fig. 5C and S20,† while for the low molecular-weight polymer-sized CFs, the surfaces are smooth.^81–83^ The wrinkled sizing layer suggests that CNDs exist under the epoxy polymer sizing surface and increase the interaction between the CFs and the epoxy sizing surface. The Raman spectra of the sized fiber samples are compared in Fig. S21.† The CND-sized CFs have a strong fluorescence excited by the laser in the Raman test, resulting in a disturbed Raman spectrum. However, the epoxy/CND-sized CFs have a normal Raman spectrum, similar to the epoxy-sized samples. Therefore, it can be inferred that all the CNDs are covered completely by epoxy in the epoxy/CND sizing process and have a strong interaction with the CFs and epoxy sizing surface, respectively.

As expected, the CNDs improve the carbon fiber/epoxy matrix interfacial interaction. The interlaminar shear strength (ILSS) of the short beams is a key indicator of the interfacial interaction of the CF composites.^84^ As shown in Fig. 5D, the ILSS of the unsized CF/epoxy short beam is 44.4 MPa and those of the epoxy-sized CFs, the CND-sized CFs and the CND/epoxy-sized CFs are 53.6 MPa, 62.3 MPa and 78.4 MPa, respectively. These results suggest that the interfacial adhesion was improved by the CNDs, in accord with the SEM analysis of the CF/epoxy composite rupture faces (Fig. S22 and 23†). Ruptures of the unsized CF sample are obvious at the interface of the CF and matrix, revealing naked CFs (Fig. S22A†). Those of the epoxy/CND-sized CFs are in the matrix, with remaining obvious epoxy fragments sticking onto the CFs (Fig. S22D†). The remaining epoxy fragments on the CFs are evidence of the strong interfacial interaction in the composites.^85^

Enhancement of the interfacial interaction in the CF composites may be mainly attributed to the large-size PAH inner structures and abundant functional groups of the CNDs, which have strong interactions with the CF and epoxy, respectively. There is a skin–core structure in the CFs, where the skin has abundant graphite crystallites. It has been claimed that the π–π interaction of the arene structure of the aromatic polymers with the CFs is strong in the composites.^86^ Strong adsorption of PAH by a graphite sheet has also been reported.^87^ The six-ring PAH has a 50 pN adhesion force per molecule with the graphene, which was measured directly by atomic force microscopy.^88^ The larger size of the PAH means more adhesion force per carbon atom.^89^ The polar group-substituted PAH should have a higher adhesion force on the graphite structure, due to dramatically increased electrostatic forces.^90^ Hence, with the PAH inner structure, abundant carboxyl groups, keto groups and hydroxyl groups, the CNDs have a high adhesion force on the CF surface due to strong π–π interaction with the surficial graphite crystallites of the CFs.

On the other hand, the abundant functional groups of the CNDs have strong chemical bonds, hydrogen bonds and van der Waals forces with the epoxy matrix.^85^ The 18.9 wt% carboxyl groups in the CNDs may form chemical bonds and hydrogen bonds with the abundant hydroxyl groups in the epoxy matrix, as illustrated in Fig. 5A. The keto groups and hydroxyl groups also have hydrogen bonds with the epoxy.^84^ Considering the van der Waals force, the CNDs have a strong interaction with the epoxy matrix. Therefore, the π–π interaction of the CND PAH structure and CF graphite surface, as well as the various bindings of the CND functional groups and epoxy matrix, result in a strong interface interaction in the CF composites.

## Conclusions

A PAH-structured carbon substance was prepared from glucose at 200 °C by hydrothermal carbonization. This is an exciting result in low-temperature hydrothermal carbonization research, which is very different from previously reported results certified by NMR. A facile and effective method was developed for large-scale fabrication of PAH CNDs with high yield, *via* hydrothermal treatment of monolithic precursor/silicone hydrogel. The formation of an insoluble large-size byproduct is inhibited in the carbonization by the nano-vessels of the methyl silicone hydrogel. Typical glucose-derived CNDs fabricated using this method can be purified facilely without dialysis, owing to the pH dependence of the CND solubility. The PAH CNDs consist of a deep carbonized structure with PAH clusters and aliphatic chains. The PAH clusters have a large fused arene structure, and the aliphatic chains contain few methyl groups and perhaps an aliphatic ring structure. Formation of the PAH structure in the CNDs has been proposed and is worth further confirmation. The CNDs have diameters of 2–5 nm and contain 18.9 wt% carboxyl groups. Application of the CNDs in the CF composite fabrication process shows obvious improvements in interlaminar shear strength, which may be attributed to the abundant functional groups of the CNDs and the strong π–π interaction of the large PAH structures in the CNDs, as well as the surficial graphite crystallites of the CFs.^88–90^

## Funding sources

This work is supported by the Special Foundation of the President of the Chinese Academy of Sciences (Tiaocaizi [2018] no. 1).

## Conflicts of interest

The authors declare no competing financial interest.

## Supplementary Material

RA-010-C9RA08128C-s001
