# Supplementary material for: Polyaromatic hydrocarbon inner-structured carbon nanodots for interfacial enhancement of carbon fiber composite
Source: RSC Adv. 2020 Jan 2;10(1):411–23. doi: 10.1039/c9ra08128c (PMC9047968; doi:10.1039/c9ra08128c)
Supplement: RA-010-C9RA08128C-s001 [file RA-010-C9RA08128C-s001.pdf]

Electronic Supplementary Material (ESI) for RSC Advances.
This journal is © The Royal Society of Chemistry 2019

SUPPORTING INFORMATION

Polyaromatic Hydrocarbon Inner-structured Carbon Nanodots for Interfacial Enhancement of Carbon Fiber Composite

Xian F. Xi, [†] *Yao Y. Li,* [‡] *Liu He** [‡]

† Zhongtian Fluorine-silicone Material Co., Ltd, Zhongtian Group

Quzhou Zhejiang 315201, P. R. China

‡ Ningbo Institute of Material Technology and Engineering, Chinese Academy of Sciences, Ningbo Zhejiang 315201, P. R. China

Correspondence should be addressed to: E-mail: heliu@nimte.ac.cn (Liu He*).

Contents

1. Experiment details 4
1.1. Preparation of Monolithic glucose silicone water gel (MGSH) 4
1.2. Preparation of CNDs by hydrothermal treatment of MGSH 4
1.3. Preparation of the hydrothermal carbon 5
1.4. Specific surface area analysis of the MGSH...5
1.5. FTIR spectra of CNDs 5
1.6. Liquid-state ^{13}C nuclear magnetic resonance of the CNDs in methanol-d. 6
1.7. Solid-state ^{13}C NMR and solid ^{1}H NMR of the CNDs 6
1.8. TEM analysis of CNDs...6
1.9. Raman spectroscopy analysis 6
1.10. XPS analysis 7
1.11. PH titration of CNDs 7
1.12. Optical characterizations of CNDs 7
1.13. SEM analysis 7
2. The simplified procedure for MGSH CNDs preparation..8
Figure S1...8
3. The N-hybrid MGSH CNDs were extracted from the silicone framework 9
Figure S2...9
4. CF/epoxy composite short beams for ASTM D2344 test..9
Figure S3...9
5. Frozen-dried MGSH and hydrothermal treated MGSH..10
Figure S4...10
6. Adsorption and desorption analysis of the frozen-dried MGSH 11
Figure S5...11
7. The pH dependence of MGSH CND solubility 11
Figure S6...11
8. Calculation of COOH content in CNDs...12
Figure S7...12
9. Liquid-state ^{13}C NMR of the CNDs..13
Figure S8...13
10. Contrast of the MGSH CND ^{13}C NMR with the classical HTC ^{13}C NMR 13
Figure S9...13
11. Contrast of the MGSH CND ^{13}C CP NMR with those of the classical GO and HTC by microwave-assisted hydrothermal processing at high temperature 14
Figure S10...14
12. Contrast of the MGSH CND ^{13}C CP NMR to those of coals with different coalification ranks 15
Figure S11...15

13. Deconvolution of the FT Raman spectra of MGSH CNDs 16
Figure S12. 16
Table S1. 16
14. Comparation the IR spectra of CNDs and graphene oxide (GO) 17
Figure S13. 17
15. XPS characterization of MGSH CNDs 18
Figure S14. 18
16. Deconvolution of the FT-IR Spectra of MGSH CNDs 18
Figure S15. 18
Table S2. 19
17. Contrast of the MGSH CND ^{13}C NMR with different framework (silica Vs silicone). 19
Figure S16. 20
18. Deconvolution of ^{13} C CP-MAS-TOSS NMR spectra of MGSH CNDs and calculation of the Average number of rings per PAH cluster 20
Figure S17. 20
Table S3 21
19. Images of the epoxy emulsion and epoxy/CND emulsion 22
Figure S18. 22
20. SEM images of the CND sized CFs 23
Figure S19. 23
Figure S20. 24
21. Raman spectra of the CF samples 25
Figure S21. 25
22. SEM images of the CFs and CF/Epoxy composite rupture faces. 25
Figure S22. 25
Figure S23. 26
23. References in ESI 27

1. Experiment details

The sodium methyl silicate solution (solid content 42 wt.%, Na_2O content 10 wt.%) was supplied by Zhejiang Zhongtian Fluorine-silicone Material Co., Ltd, Zhejiang, China. Other chemicals were purchased from Sinopharm Chemical Reagent Company and used as received without any further purification. Deionized water (18.2 MΩ cm @25 °C) was used in all experiments.

1.1. Preparation of Monolithic glucose silicone water gel (MGSH)

First, 250 g 40 wt.% glucose solution was prepared by dissolving 100 g of D-glucose monohydrate in 150 g of deionized water. Next, 72 g of HCl solution (18 wt.%, 36.5 wt.% HCl was diluted by deionized water water) was mixed with the glucose solution. And then, the HCl-glucose solution and 180 g of sodium methyl-silicate solution (solid content 42 wt.%, Na_2O content 10 wt.%) were poured into a 500-ml beaker with vigorous stirring for 30 s. finally, the semilucent blue MGSH formed with a glucose content of 18.3 wt.%. The appearance and porosity of MGSH is dependent on the quantity of HCl added. In each sample, 10 g MGSH was cut and soaked in 3000 ml deionized water for 48 h and Frozen-dried for BET characterization (Figure S3). All the CNDs discussed in the paper refer to CNDs made from the MGSH prepared using the above procedures, unless indicated otherwise. This MGSH was named ***sample 1***.

For comparation, the monolithic silicone hydrogel with 10% glucose and 10% ethanediamine was prepared by the same method and named ***sample 2***.

1.2. Preparation of CNDs by hydrothermal treatment of MGSH

The MGSHs and other N-hybrid MGSHs were sealed in beakers and then sealed in a 2000 ml Teflon-lined with 50 ml of water, which maintained the vapor pressure balance. After heating at 200 °C for 24 h, the semilucent blue MGSHs transformed into black methyl silicone hydrogel with glucose-derived CNDs or brown methyl silicone hydrogel with N-hybrid CNDs. methyl silicone hydrogel with glucose-derived CNDs was broken into black slurry, and the pH was adjusted to 1 with HCl solution. The slurry was washed and filtered 5 times with deionized water, and the filter residue was added into 500 g of 40 wt.% HF solution in a polypropylene beaker

very slowly with stir. After reaction and vaporization of the silicone for 48 h, the CNDs were washed 5 times by 20 times weight deionized water. The weight of the wet CNDs varies 138–145 g (repeated 6 times), and part of the CNDs was dried at room temperature in a vacuum oven for 24 h for the tests. The wet CNDs included 75% water and 25% CNDs approximately. The yield is 65% (calculated by carbon atoms). the CND solutions were prepared from the wet CNDs. The first washed water was collected for fluorescence characterization in **Figure S1A**. The hydrazine-hydrate-modified MGSH CNDs were prepared by reflux of hydrazine hydrate and wet as-synthesized MGSH CNDs for 24 h; the FL emission and excitation are shown in **Figure 5B.**

The hydrothermal-treated-MGSH slurry was treated with insufficient HF solution, and then was washed with above method. The remain CNDs dried at room temperature in a vacuum oven for 24 h and was named **CNDs@silicone**. After hydrothermal carbonization, the ***sample 2*** were first extracted using deionized water and then extracted using DMF, named **Figure 5C** and **Figure 5D** respectively.

1.3. Preparation of the hydrothermal carbon

According the report, [1] 600 g glucose water solution with a content of 10 wt.% was sealed in a glass tube, and then sealed in a 2000 ml Teflon-lined with 50 ml of water, which maintained the vapor pressure balance. After heating at 200 °C for 24 h, the brown-black sediments formed. After washing and filtration with deionized water 6 times, about 24 g remaining sediments were dried in vacuum oven at 120 ℃ for 24 h before ^{13}C NMR test.

1.4. Specific surface area analysis of the MGSH

The specific surface area and pore size of the samples were determined by physical adsorption (N2 at -77.7 K) using a Micrometrics ASAP 2020 HD88 physisorption analyzer after sample outgassing at 393 K under vacuum for 5 h.

1.5. FTIR spectra of CNDs

FT-IR spectra were obtained using a Nicolet 6700 FT-IR spectrometer, Thermo Scientific Co., Ltd, American. The dried CND powder samples were blended with the

pre-dried potassium bromide powder at a weight ratio of 0.15. After grinding, the mixtures were pressed into pellets and tested.

1.6. Liquid-state ^{13}C nuclear magnetic resonance of the CNDs in methanol-d.

Liquid-state ^{13}C NMR spectrum was performed on a Bruker AVANCE III 400 in deuterated methanol at a resonance frequency of 400 M Hz. The concentration of the CNDs in the deuterated methanol solution is about 0.3 wt.%.

1.7. Solid-state ^{13}C NMR and solid ^{1}H NMR of the CNDs

The ^{13}C (H) CP TOSS MAS spectra were recorded on a Bruker AVANCE III 400 WB spectrometer equipped with a 4-mm standard bore CP MAS probe head with an X channel tuned to 100.62 MHz for ^{13}C and the other channel tuned to 400.18 MHz for broadband ^{1}H decoupling; a magnetic field of 9.39 T at 297 K was used. The dried and finely powdered CNDs were packed in the ZrO_2 rotor closed with a Kel-F cap and were spun at 8 kHz. The experiments were conducted using a contact time of 2 ms. A total of 2000 scans were recorded with 6-s recycle delay for each sample. All the ^{13}C CP MAS chemical shifts are referenced to the resonances of an adamantane ($C_{10}H_{16}$) standard (δCH_2=38.4 ppm).

1.8. TEM analysis of CNDs

TEM analysis of the samples was then performed using a Tecnai F20 microscope. First, 0.002 g of the **wet** CNDs (25wt.%, as shown in **Figure S1**) were added to 10 ml of isopropanol (HLPC Plus, Sigma-Aldrich) and then treated by ultrasound for 15 min. The ultrathin carbon supporting films for the TEM were placed on a piece of fresh filter paper, and a drop of the CND solution was added. Then, the carbon supporting films with samples were dried in a vacuum oven at 80 °C before testing.

1.9. Raman spectroscopy analysis

Raman spectroscopy analysis was performed on a Renishaw inVia Reflex confocal Raman microscope at 542 nm excitation wavelength. Before testing, the CND sample was heat-treated at 160 °C for 12 h in a vacuum oven to eliminate fluorescence. Deconvolution of the spectra was performed by assuming Gaussian

peaks to describe bands. The data treatment was performed using Origin Pro 2015 software. In fact, a serious of lights (542 nm-1030 nm) were tried, and the result shows no obvious difference. Only the 542 nm excitation results were saved.

The unsized and sized CFs were test by Renishaw inVia Reflex confocal Raman microscope at 542 nm excitation wavelength directly, without heat-treatment. The CND sized CF has a disturbed Raman spectrum, due to the CND fluorescent.

1.10. XPS analysis

XPS analysis was performed using an AXIS ULTRA X-ray photoelectron spectrometer using an Mg K X-ray source (1253.6 eV) operated at 14 kV and 300 W with an emission current of 25 mA. Survey scans were collected from 0 to 1100 eV. Casa XPS instrument software was used for the deconvolution of the XPS spectra. For calibration purposes, the C 1s electron binding energy from adsorbed ubiquitous organic material was referenced at 284.6 eV. Different functional groups were assigned using reported C 1s chemical shifts in various organic compounds. The relative amounts of these groups were estimated from respective areas of assumed Gaussian curves.

1.11. PH titration of CNDs

First, 1.2 g of **wet** CNDs (25 wt.%) was dissolved in 148.5 g of ethyl alcohol water solution (50 wt.%), and then, we obtained the CND solution (0.2 wt.%). Next, 10.9 g of the CND solution was added to 15 ml of standard NaOH solution (0.0493 M) and stirred for 5 min. The titration was finished with HCl solution (0.0489 M) in a 905 Titrando.

1.12. Optical characterizations of CNDs

PL emission and excitation spectra were obtained using a Hitachi F-4600 spectrophotometer under ambient conditions. The 30 ppm CND water solutions were scanned using a slit width of 5 nm from 280 to 580 nm, and the emission intensities from 380 to 700 nm for each scan were recorded.

1.13. SEM analysis

The wet CNDs were dissolved in the ethyl alcohol with a concentration of 0.1 wt.%. The solutions were dropped on the silicon wafer and dried in vacuum oven. The dried samples were examined using scanning electron microscopy (SEM; Hitachi 4800, Japan) at an accelerating voltage of 5–8 kV. The Carbon fiber (CF) samples were characterized by the same method. The rupture faces of CF/epoxy composite beams were characterized by the Hitachi TM1000 SEM, with an accelerating voltage of 15 kV. All the samples were sprayed platinum before test.

2. The simplified procedure for MGSH CNDs preparation

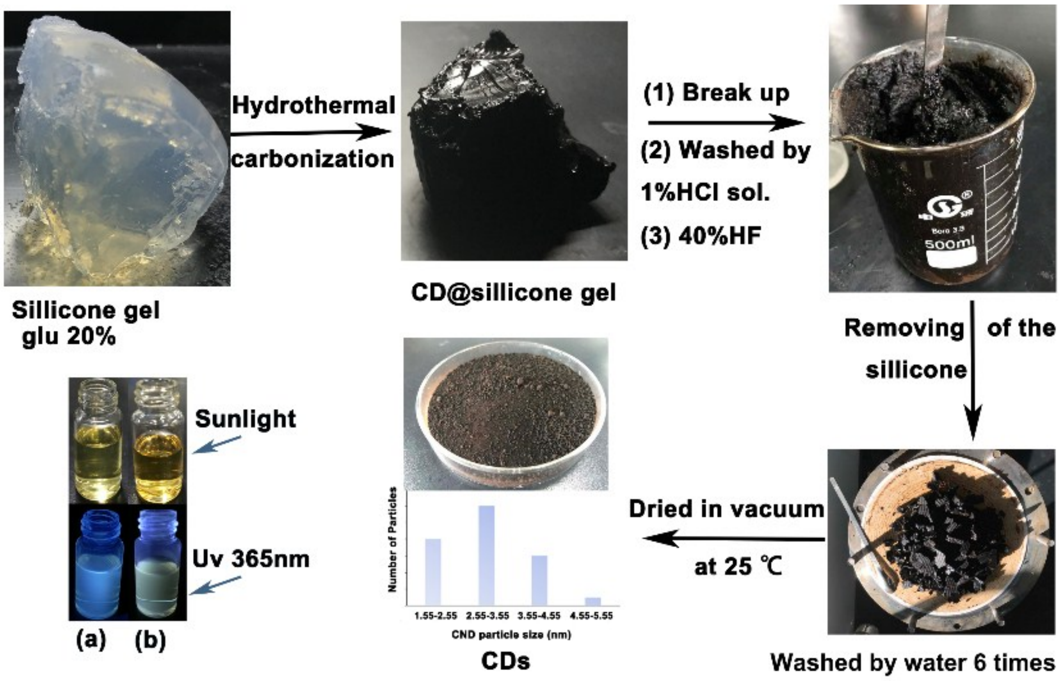

Figure S1. Procedures for preparation of CNDs via hydrothermal treatment of the Monolithic glucose silicone hydrogel. The removal of the silicone by HF (aq.) was performed in an ugly polypropylene beaker, not in this glass beaker. CNDs in the wastewater in purification process (A). Remaining CNDs water solution with a concentration of about 50 ppm (B). The size distribution of carbon nanodots was calculated according to the TEM image (Figure 4G).

3. **The N-hybrid MGSH CNDs were extracted from the silicone framework**

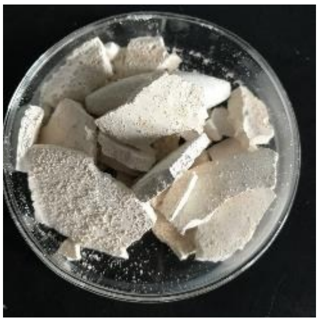

Figure S2. The remained silicone framework after the N-hybrid CNDs were extracted by DMF from the CND slurry, indicating N-hybrid CNDs have a very high solubility in DMF and there are no nano-carbon particles anchored on the framework.

4. **CF/epoxy composite short beams for ASTM D2344 test**

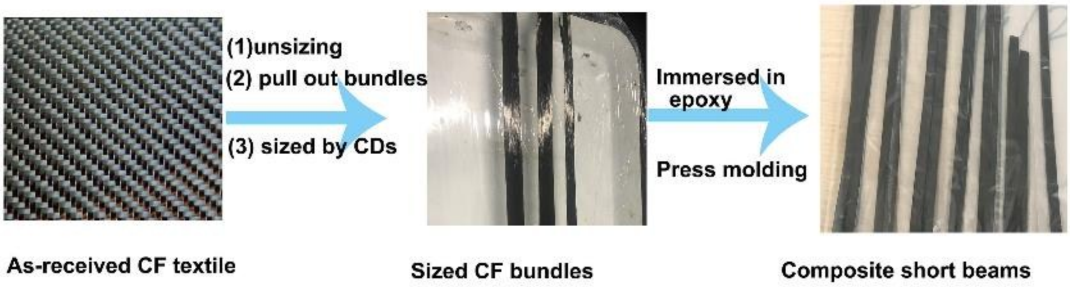

Figure S3. Preparation of the CF composite beams for interlaminar shear strength tests

5. Frozen-dried MGSH and hydrothermal treated MGSH

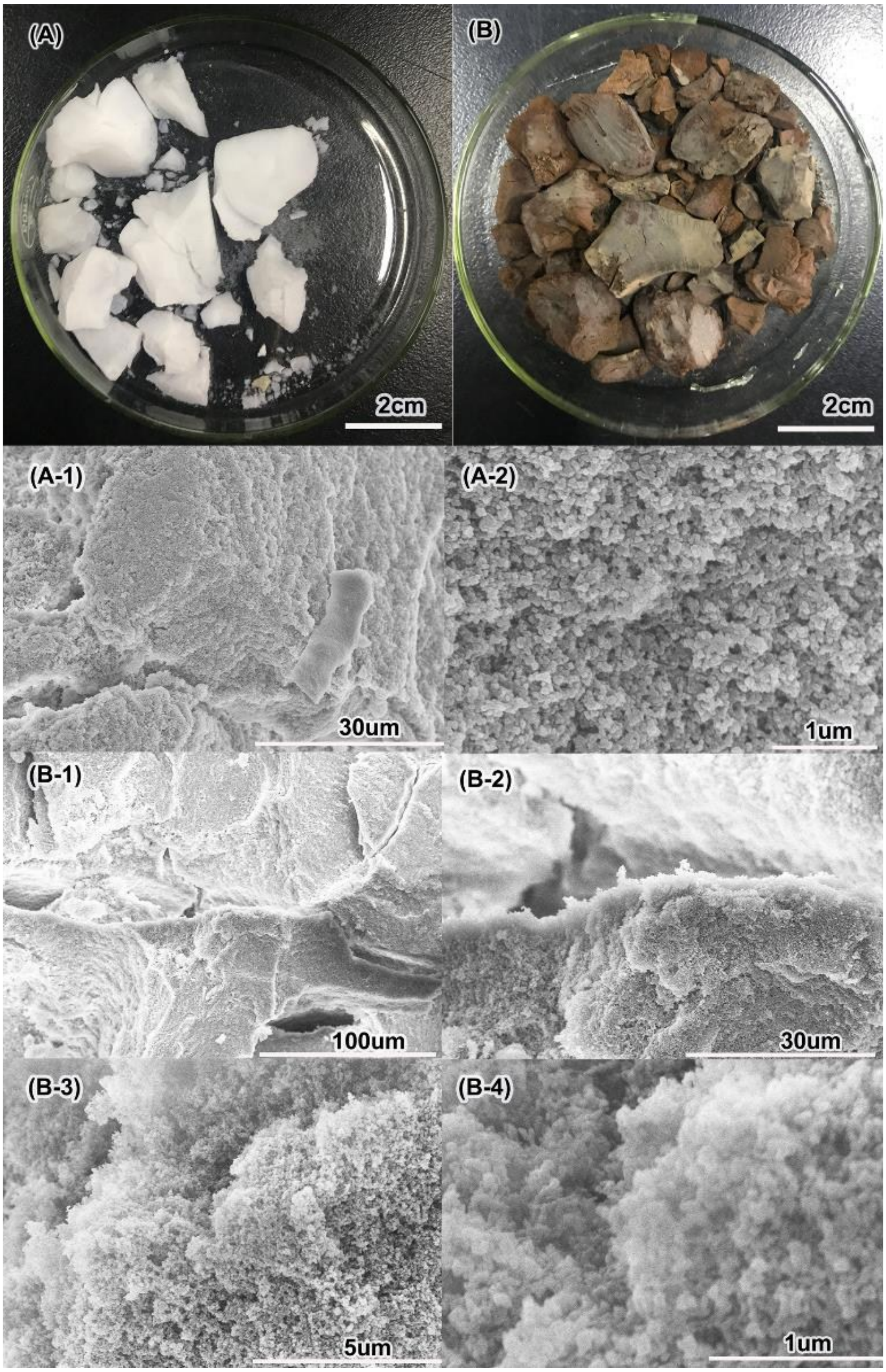

Figure S4. Frozen-dried MGSH (A, A1-A2) and Frozen-dried hydrothermal-treated-MGSH (B, B1-B4), which were soaked in 3000 ml deionized water for 48 h firstly. They are aerogel. Images of A1-A4, B1-2 are captured by SEM Hitachi 4800.

6. Adsorption and desorption analysis of the frozen-dried MGSH

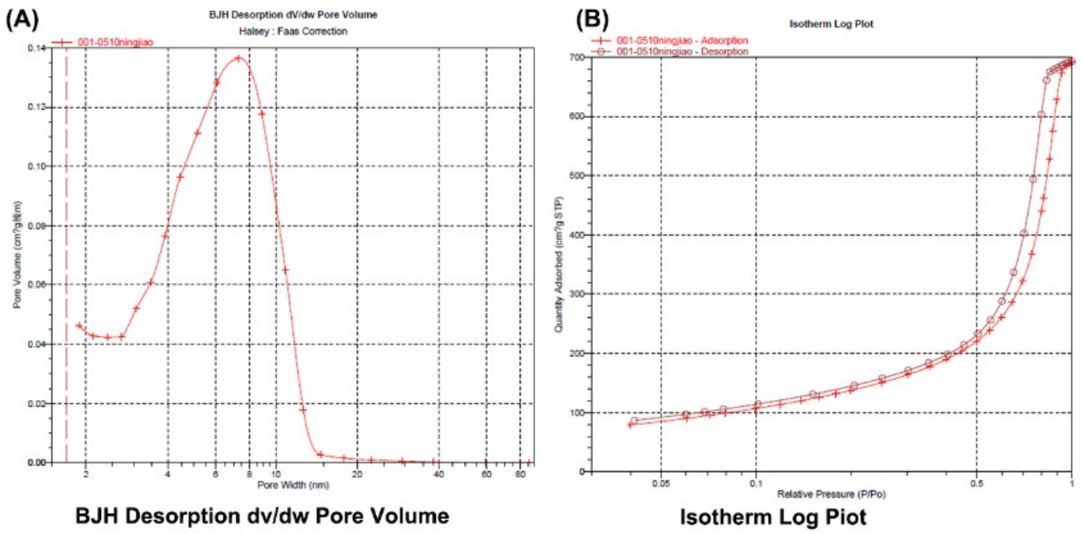

Figure S5. Adsorption and desorption analysis of the frozen-dried MGSH

7. The pH dependence of MGSH CND solubility

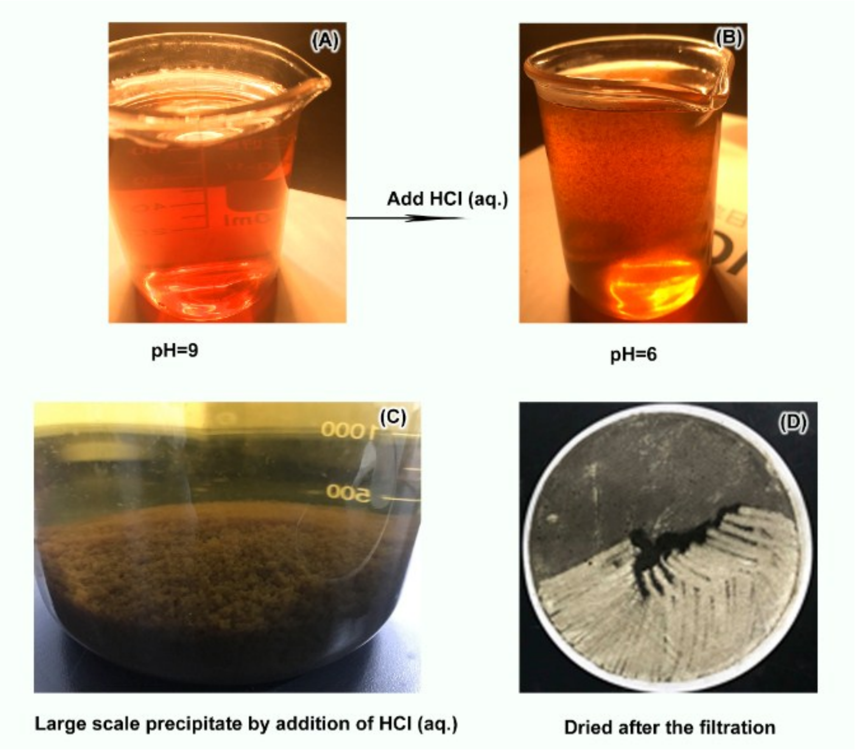

Figure S6. The pH dependence of MGSH CND solubility. The CND solution at pH=9 (A), sediments forms after addition of HCl solution (B), 3000 ml CNDs saturated solution yielded about 300 cm^3 sediments after altering the solution pH (C), the 300 cm^3 sediments weight 1.5 g after drying (D).

8. Calculation of COOH content in CNDs

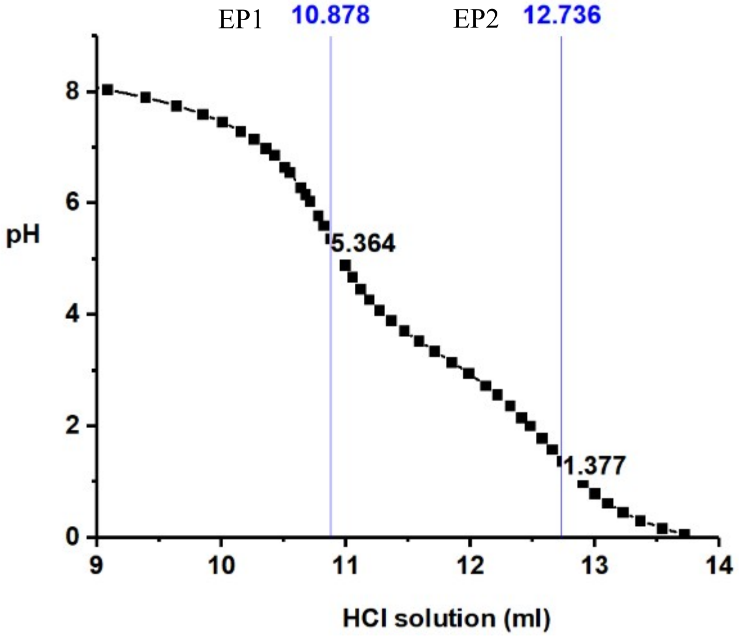

Figure S7. Titration curve of MGSH CNDs.

The endpoint (EP) is determined using the machine 905 Titrando. EP1 is the endpoint of the NaOH in the solution, and EP2 is the endpoint of the carboxylate of CNDs. Therefore, the HCl consumed by the carboxylate in the CNDs is

12.736 ml − 10.878 m l=1.858 ml

We obtained a COOH amount of $1.858\ ml \times M$.

COOH number is 9.16×10^{-5}mole, weight 0.00412 g

CND weight is $10.9\ g \times 0.20\ wt.\%$=0.0218 g

Therefore, we obtained a COOH content (wt.%) in the CNDs of $0.00412\ g \div 0.0218\ g \times 100\%$=18.9%.

9. Liquid-state ^{13}C NMR of the CNDs.

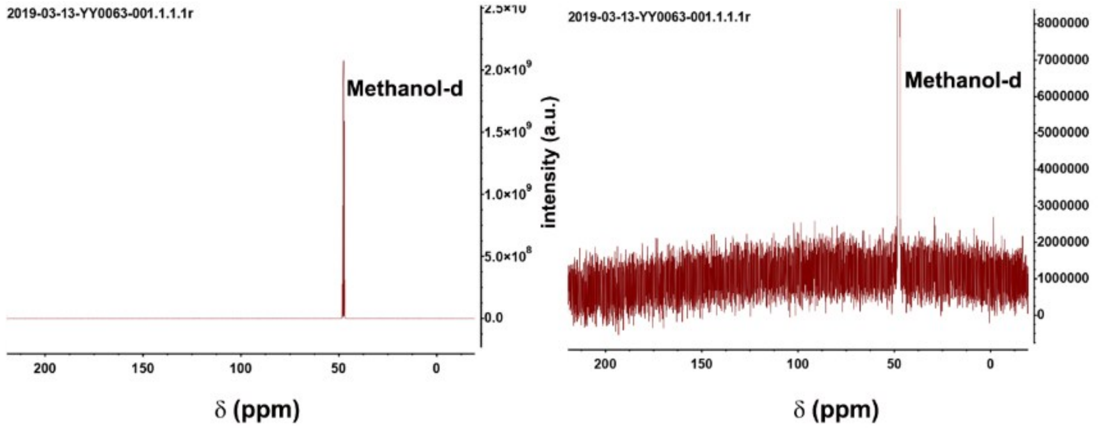

Figure S8. Liquid-state ^{13}C NMR of the MGSH CNDs. The CND concentration in the deuterated methanol solution is 0.3 wt.%. There is no any signal, suggesting that the species involved are particles (that is, a condensed phase) and not dispersed molecules.

10. Contrast of the MGSH CND ^{13}C NMR with the classical HTC ^{13}C NMR

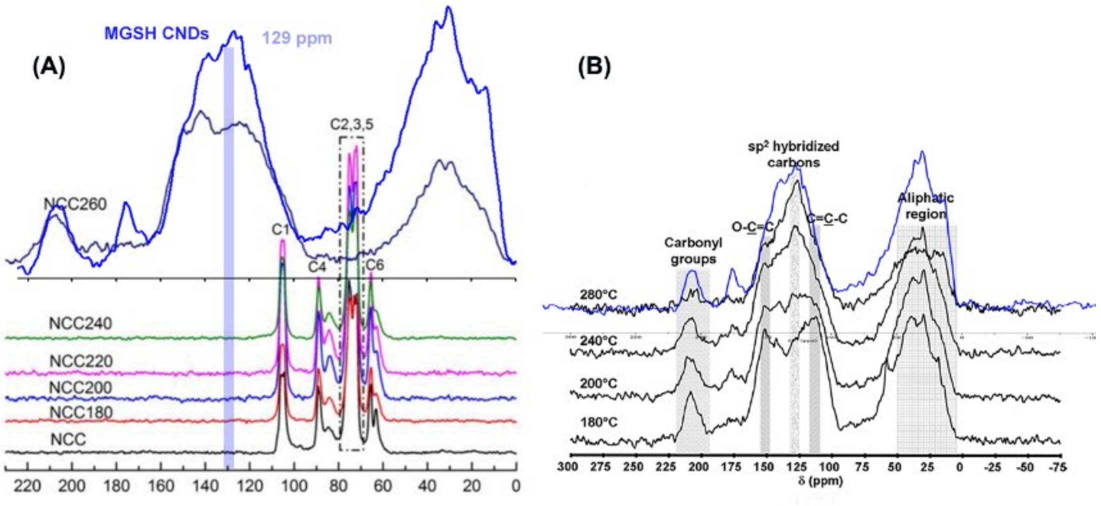

Figure S9. Contrast of the MGSH CND ^{13}C NMR with the classical HTC ^{13}C NMR. There is no peak at 150 ppm in the MGSH CND ^{13}C NMR (blue line). All the samples were hydrothermal treated 24 h at different temperature. (A); Contrast of the MGSH CND ^{13}C NMR with the hydrothermal nanocrystalline cellulose carbon dot (NCC) ^{13}C NMR. The number after NCC is the temperature of the NCC preparation (B). The figure was reproduced with permission, [2, 3] Copyright Year 2012, Royal Society of Chemistry.

11. Contrast of the MGSH CND ^{13}C CP NMR with those of the classical GO and HTC by microwave-assisted hydrothermal processing at high temperature

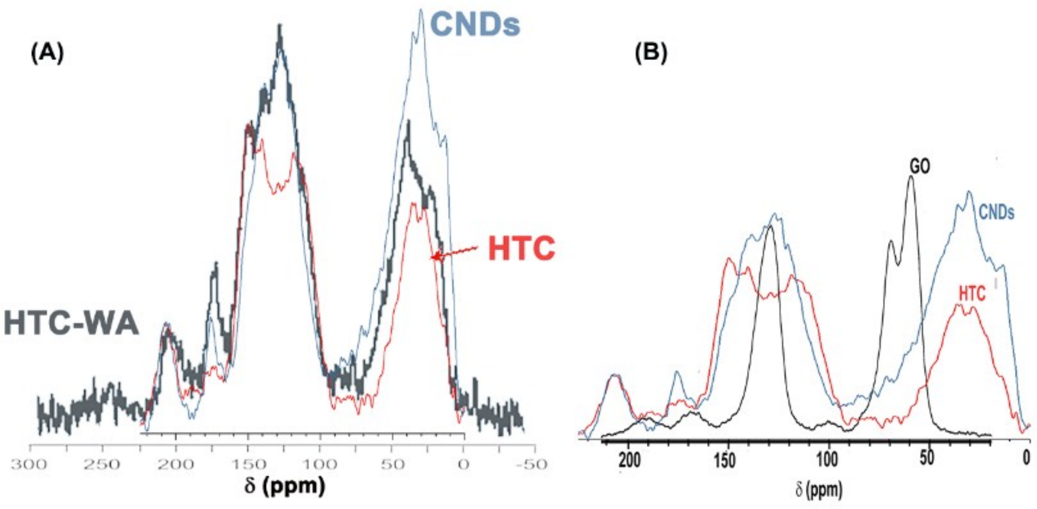

Figure S10. Comparation of the ^{13}C CP NMR spectra: CNDs (blue line); HTC (red line); HTC by microwave microwave-assisted hydrothermal processing at 250 ℃ 10h (black line). The figure was reproduced with permission, [4] Copyright Year 2011, New York Intech., (A); Comparation of the ^{13}C CP NMR spectra: (CNDs blue; HTC, red; GO black) The GO ^{13}C NMR spectrum was reproduced with permission, [5] Copyright Year 2008, Science (B).

12. Contrast of the MGSH CND ^{13}C CP NMR to those of coals with different coalification ranks

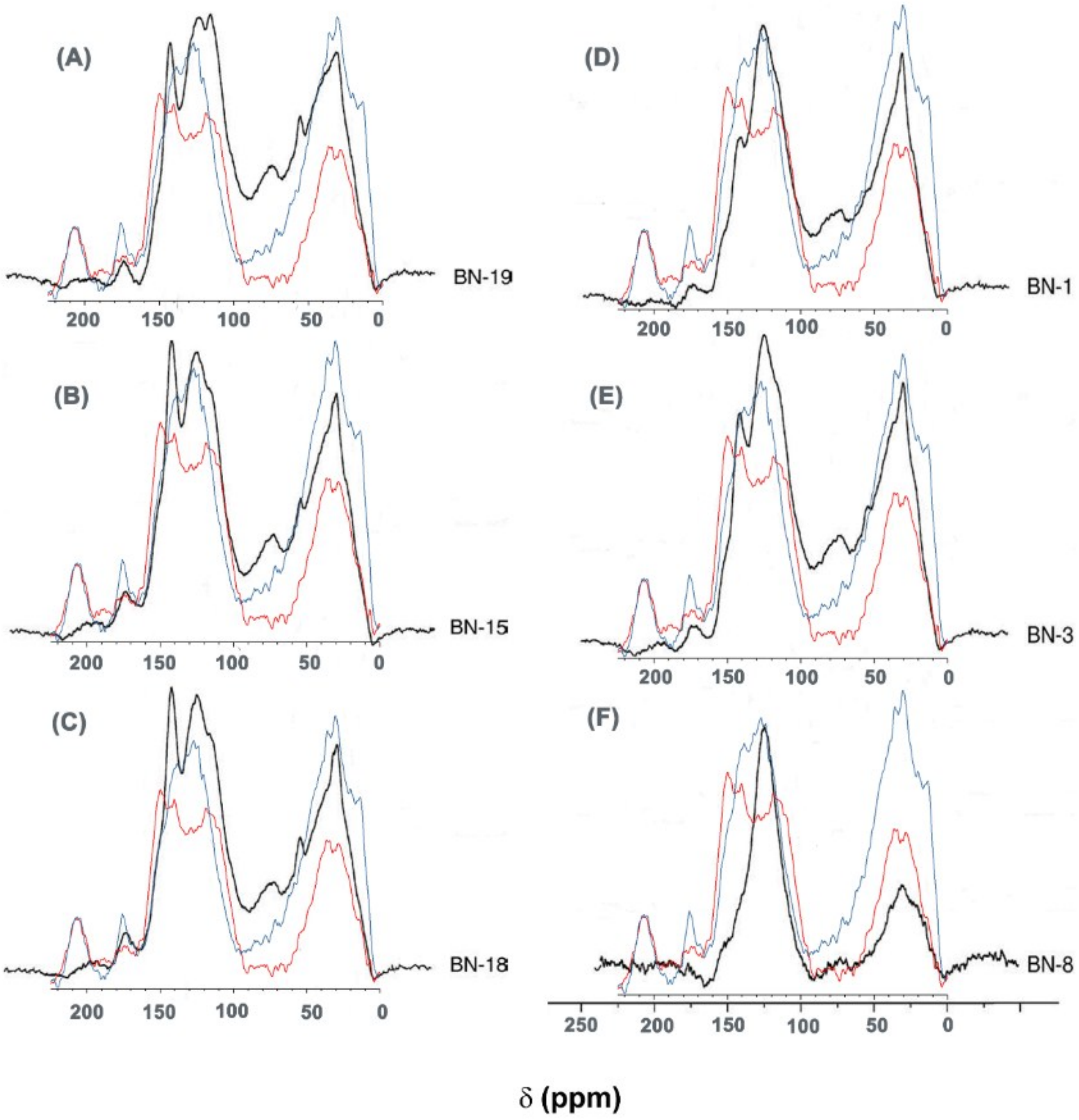

Figure S11. Comparation of the ^{13}C CP NMR spectra: the Inertinite-rich coal (F); the lignite with different coalification ranks: A, B, C, D, E. The CNDs (blue line); HTC (red line); the lignite and coal (black line). The ^{13}C NMR spectra of BN-1, BN-3, BN-8, BN-15, BN-18 and BN-19 were reproduced with permission, [6] Copyright Year 2010, Elsevier B.V.

13. Deconvolution of the FT Raman spectra of MGSH CNDs

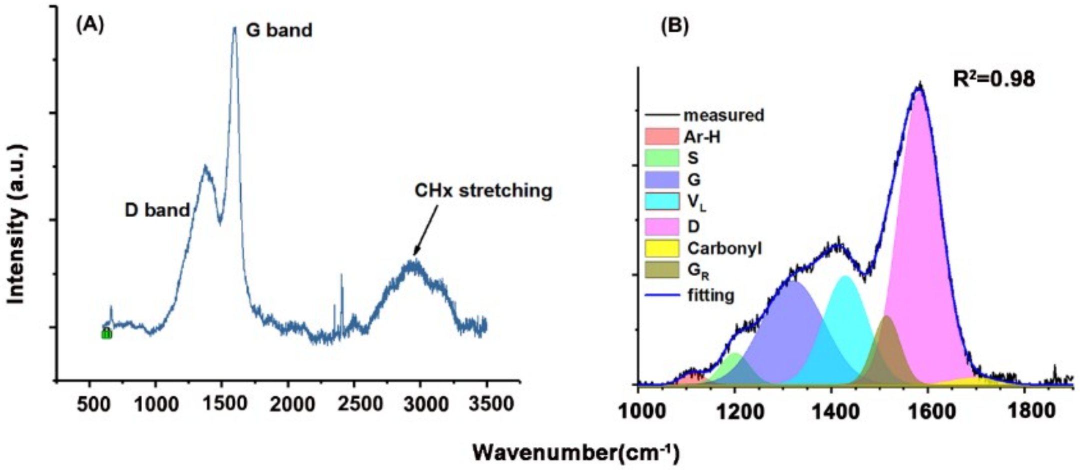

Figure S12. Raman spectrum of the CNDs (A), curve fitting in the 1000–1800 cm^{-1} band of the CND Raman spectrum (B). The samples were pretreated to eliminate fluorescent before test.

Table S1. Curve Fitting of the 1000–1800 cm^{-1} Band of the CND Raman Spectrum

Peaks	Methods	Area	Wavenumber (cm^{-1})	FWHM	height	Area %
Ar-H	Gauss	34418	1113	58	562	1.1
S	Gauss	108292	1200	70	1453	2.4
D	Gauss	762791	**1320**	151	4752	**23.9**
V_L	Gauss	569653	1428	108	4969	17.8
G_R	Gauss	222827	1514	66	3151	7.0
G	Gauss	1456910	**1583**	102	13339	**45.7**
carbonyl	Gauss	38364	1699	112	323	1.2

The G band occurs at 1583 cm^{-1}, the D band occurs at 1320 cm^{-1}, and the D/G intensity is 23.9/45.7= 0.52.

14. Comparation the IR spectra of CNDs and graphene oxide (GO)

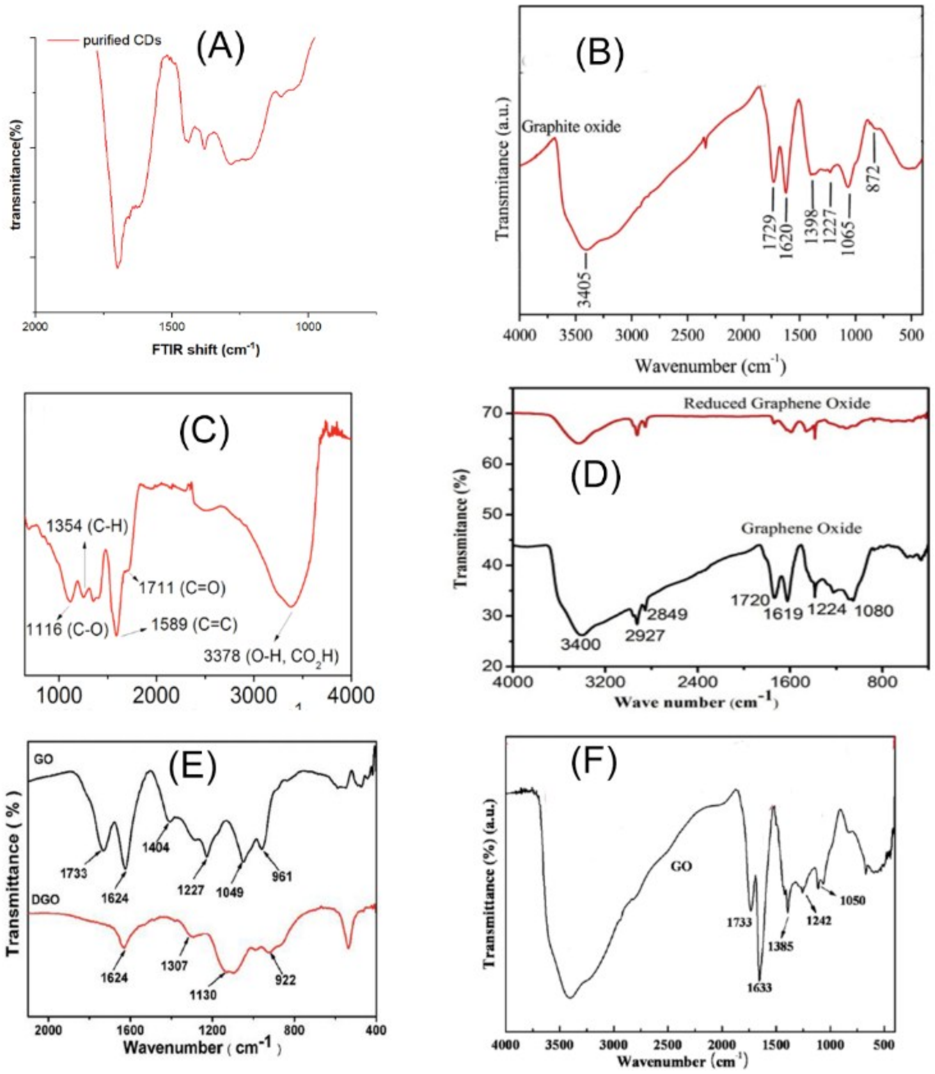

Figure S13. the 1000-1500 cm^{-1} IR spectra of CNDs in this work (A), GO (B), [7] GO (C), [8] GO (D), [9] GO (E), [10] GO (F). [11] The copyrights belong to their publishing company.

15. XPS characterization of MGSH CNDs

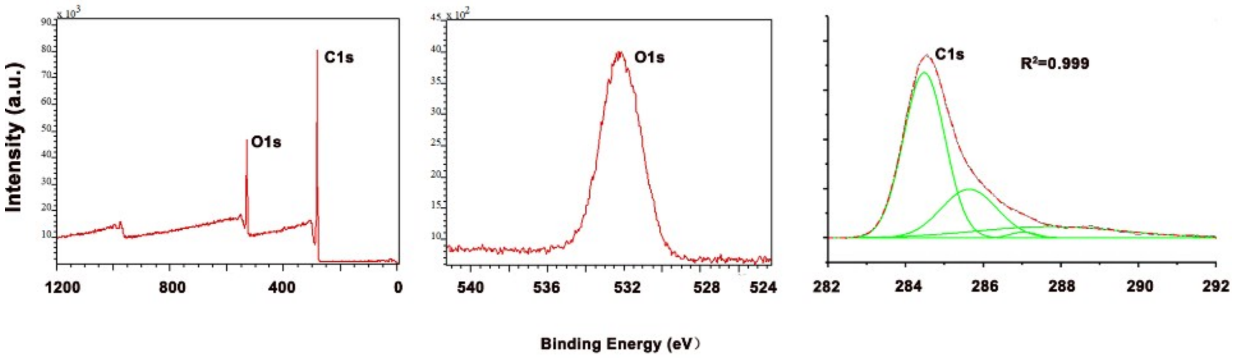

Figure S14. XPS spectra of CNDs (A), O1s fine spectra of CND XPS (B), curve fitting of carbon nanodot C1s XPS spectra.

The XPS is widely used for CND characterization. The C and O atom contents were 79 wt.% and 21 wt.%, respectively. This technique is imprecise, contrasting with the elemental analysis using a CHOSN analytical instrument.

16. Deconvolution of the FT-IR Spectra of MGSH CNDs

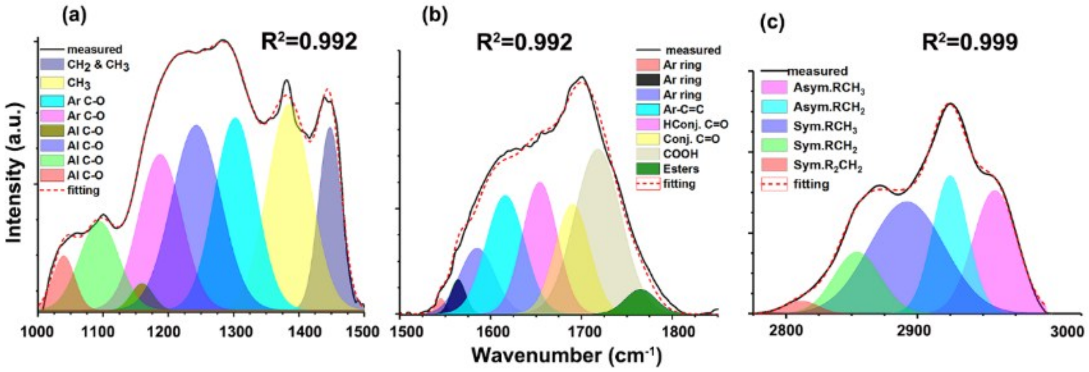

Figure S15. Curve fitting of the CND IR spectra in the range of 1000–1500 cm^{-1} (A), 1500–1850 cm^{-1} (B), and 2750–3000 cm^{-1} (C).

Table S2. Curve Fitting of FTIR Spectra of MGSH CNDs

Figures	Peaks	Methods	Area	Wavenumber (cm^{-1})	FWHM	height	Area %
A) Curve fitting of the band from 1000 to 1500 cm^{-1}	Al-C-O	Gaussian	0.80	1039	40	0.019	2.8
	Al-C-O	Gaussian	2.35	1093	71	0.031	8.2
	Al-C-O	Gaussian	0.41	1159	40	0.010	1.4
	Al-C-O	Gaussian	4.55	1187	78	0.055	15.9
	Ar-C-O	Gaussian	6.13	1243	89	0.065	21.5
	Ar-C-O	Gaussian	5.45	1302	76	0.068	19.1
	OH	Gaussian	6.04	1384	78	0.072	21.2
	CH_3 &CH_2	Gaussian	2.81	1447	41	0.064	9.9
B) Curve fitting of the band from 1500 to 1800 cm^{-1}	Ar ring	Gaussian	0.2	1545	12.6	0.014	0.6
	Ar ring	Gaussian	0.7	1564	19.9	0.033	2.2
	Ar ring	Gaussian	3.0	1585	45	0.062	9.4
	Ar ring	Gaussian	6.0	1616	50	0.112	18.8
	High-conjugated C=O	Gaussian	6.0	1654	45	0.125	18.8
	Conjugated C=O	Gaussian	5.0	1690	45	0.104	15.7
	COOH	Gaussian	10.0	1718	60	0.157	31.3
	Esters	Gaussian	1.0	1765	40	0.023	3.1
C) Curve fitting of the band from 2800 to 3000 cm^{-1}	CH_2 sym.	Gaussian	0.12	2812	29.5	0.00389	1.90
	CH_2 sym.	Gaussian	0.84	2854	41.6	0.01905	13.1
	CH_3 sym.	Gaussian	2.48	2892	67.3	0.03464	38.4
	CH_2 Asym.	Gaussian	1.44	2925	31.7	0.04266	22.3
	CH_3 Asym.	Gaussian	1.58	2959	39.0	0.03811	24.3

*The curve fittings were carried out according the structure researches of lignite. [12,13]

17. Contrast of the MGSH CND ^{13}C NMR with different framework (silica Vs silicone).

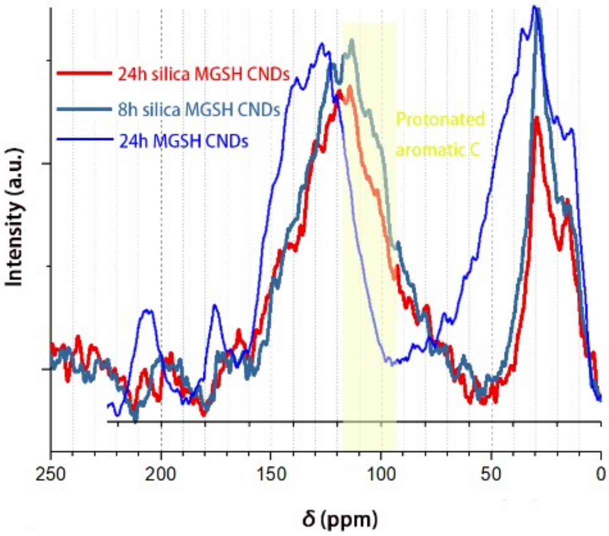

Figure S16. MGSH CND 13C NMR with different framework. The blue line is the MGSH CNDs with a silicone framework; the red line is the silica framework. The MGSH CNDs have a 128 ppm arene peak and the silica framework MGSH CNDs have a 113 ppm arene peak.

18. Deconvolution of [13] C CP-MAS-TOSS NMR spectra of MGSH CNDs and calculation of the Average number of rings per PAH cluster

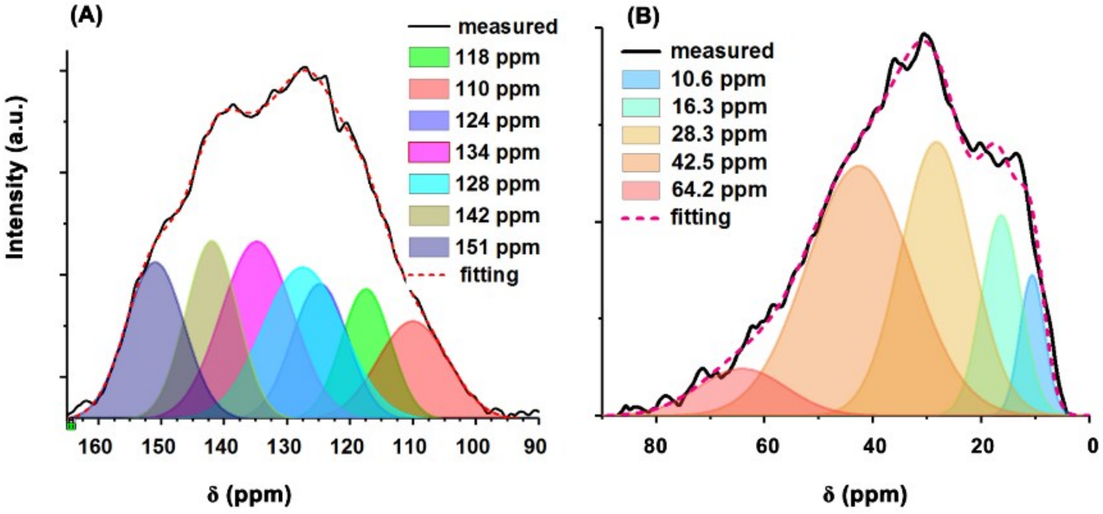

Figure S17. Curve fitting of the CND ^{13}C NMR spectra in the range of 90–160 ppm (A, just Figure 2C), and 0-90 ppm (B).

The further structure characteristics were deduced using the Solum method, [14], [15] which has been wildly used in coal chemical structure research. The protonated aromatic carbon $f_a{}^{H}$ peak always overlaps with the aromatic bridgehead and inner carbon $f_a{}^{B}$ peak in the ^{13}C CP MAS NMR spectrum; therefore, it is very difficult to calculate the $f_a{}^{H}$ using curve fitting.[16] In the structure analysis of coal, $f_a{}^{H}$ is usually inferred using ^{13}C DD NMR or ^{13}C DP NMR.[17-19] Unlike the complex organic and inorganic composition of mineral coal, there are mainly C, H, and O elements in the composition of CNDs. Therefore, ultimate analysis of the CNDs can be used to calculate the $f_a{}^{H}$ more accurately. For 100 g of the CNDs, we were able to obtain the following result:

$$aromatic\ H\ mol = 0.41 - \frac{2.68}{R+1}$$

Table S3. Structural Parameters of MGSH CNDs.

Solid state ^{13}C NMR structural parameters			
structural parameters	Curve fitting [20-22]		result
	δ□(ppm)	Content%	
aromatic carbon, $f_a = f_a' + f_a^C$	90-220	-	0.51
carbonyl, $f_a^C = f_a^O + f_a^{OO}$	165-220	-	0.09
aldehydes and ketones, f_a^O	205	0.048	0.048
carbonyl, f_a^{OO}	175	0.042	0.042
aromatic carbon, carbonyl subtracted, f_a'	90-165	0.42	0.42
protonated aromatic carbon, f_a^H	90-125	-	$0.076 - \frac{0.49}{R+1}$, $R \geqslant 5.53$
non-protonated aromatic carbon, $f_a^N = f_a^P + f_a^S + f_a^B$	120-165	-	0.344+0.494/(R+1), $R \geqslant 5.53$
aromatic carbon with oxygen attachment (phenolic), f_a^P	151	0.060	0.060
aromatic carbon with alkyl attachment, f_a^S	141	0.144	0.144
aromatic bridgehead and inner carbon, f_a^B	120-135	-	0.14+0.494/(R+1), $R \geqslant 5.53$
aliphatic carbon, f_{al}	0-90	0.49	0.49
aliphatic CH and CH_2, f_{al}^H	28.3 & 42.5	0.311	0.311
aliphatic CH_3, f_{al}^*	10.6 & 16.3	0.075	0.075
aliphatic with oxygen attachment, f_{al}^O	64.2	0.075	0.075
aromatic bridgehead carbons, χ_b	-	-	0.333+1.18/(R+1), R ≥5.5
$\chi_{b\ min.}$	-	-	0.33, if $R \to \infty$, f_a^H=0. 179
$\chi_{b\ max.}$	-	-	0.52, if R=5.53, f_a^H=0
average number of carbons per PAH cluster, C_{Min}	-	-	16, if $R \to \infty$, f_a^H=0. 179
average number of carbons per PAH cluster, C_{Max}	-	-	24, if R=5.53, f_a^H=0
average number of rings per PAH cluster, C_{Min}	-	-	3
average number of rings per PAH cluster, C_{Max}	-	-	8

From the elemental analysis data (**Table 1**) and C atom ratios obtained from the ^{13}C NMR spectra (**Figure 2B**), we obtained the following results:

$$aromatic\ Cweight = 100g \times 65.62\%$$

$$aromatic\ C\ mol = \frac{aromatic\ Cweight}{12g/mol}$$

Therefore, we obtained $aromatic\ C\ mol = 5.34$ in 100 g of CNDs.

$$f_a^H = \frac{aromatic\ H\ mol}{aromatic\ C\ mol}$$

From this result, we obtained $f_a^H = 0.076 - \frac{0.49}{R+1}$.

According to previous reports, [15,23] the aromatic carbon, $f_{a,}$ carbonyl, f_a^{C}, aldehydes and ketones, f_a^{O}, carbonyl, f_a^{OO}, aromatic carbon with oxygen attachment (phenolic) f_a^{P}, aromatic carbon with alkyl attachment, f_a^{S}, aliphatic carbon f_{al}, aliphatic CH and CH_2 f_{al}^{H}, aliphatic CH_3 and non-protonated carbon, f_{al}^{*}, and aliphatic with oxygen attachment f_{al}^{O} were calculated based the result of curve fitting of the ^{13}C CP MAS TOSS NMR spectra.

The main peak of the aliphatic C occurs near 33 ppm, which differs from the main peak near 50–70 ppm for pyrolytic carbon because of the remaining carbohydrate structure. [24] The aliphatic C structure is similar that of hydrothermal carbon spheres and brown coals. [2] The average number of rings per PHA cluster inferred by NMR is still not very accurate because of the technology limits.

19. Images of the epoxy emulsion and epoxy/CND emulsion

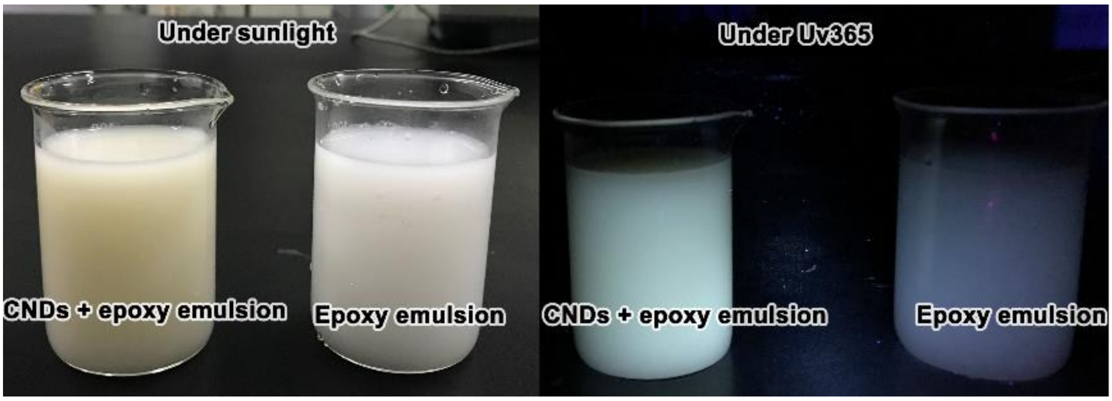

Figure S18. Images of the epoxy emulsion sizing and epoxy/CND sizing.

20. SEM images of the CND sized CFs

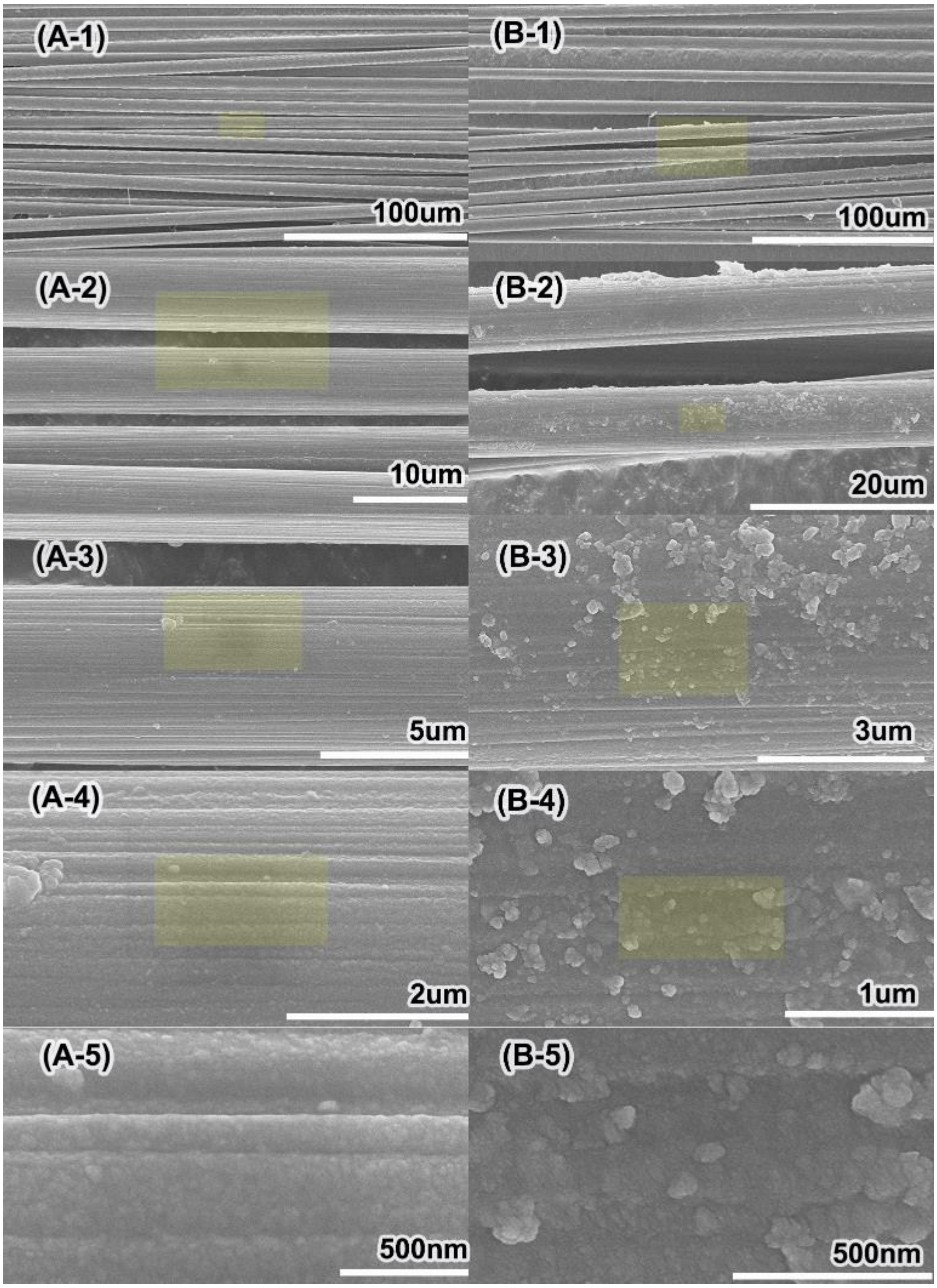

Figure S19. SEM images of the CND sized CFs. A1-5 are CDs sized by 0.01wt.% CND solution. B1-5 are sized by 0.1 wt.% CND solution.

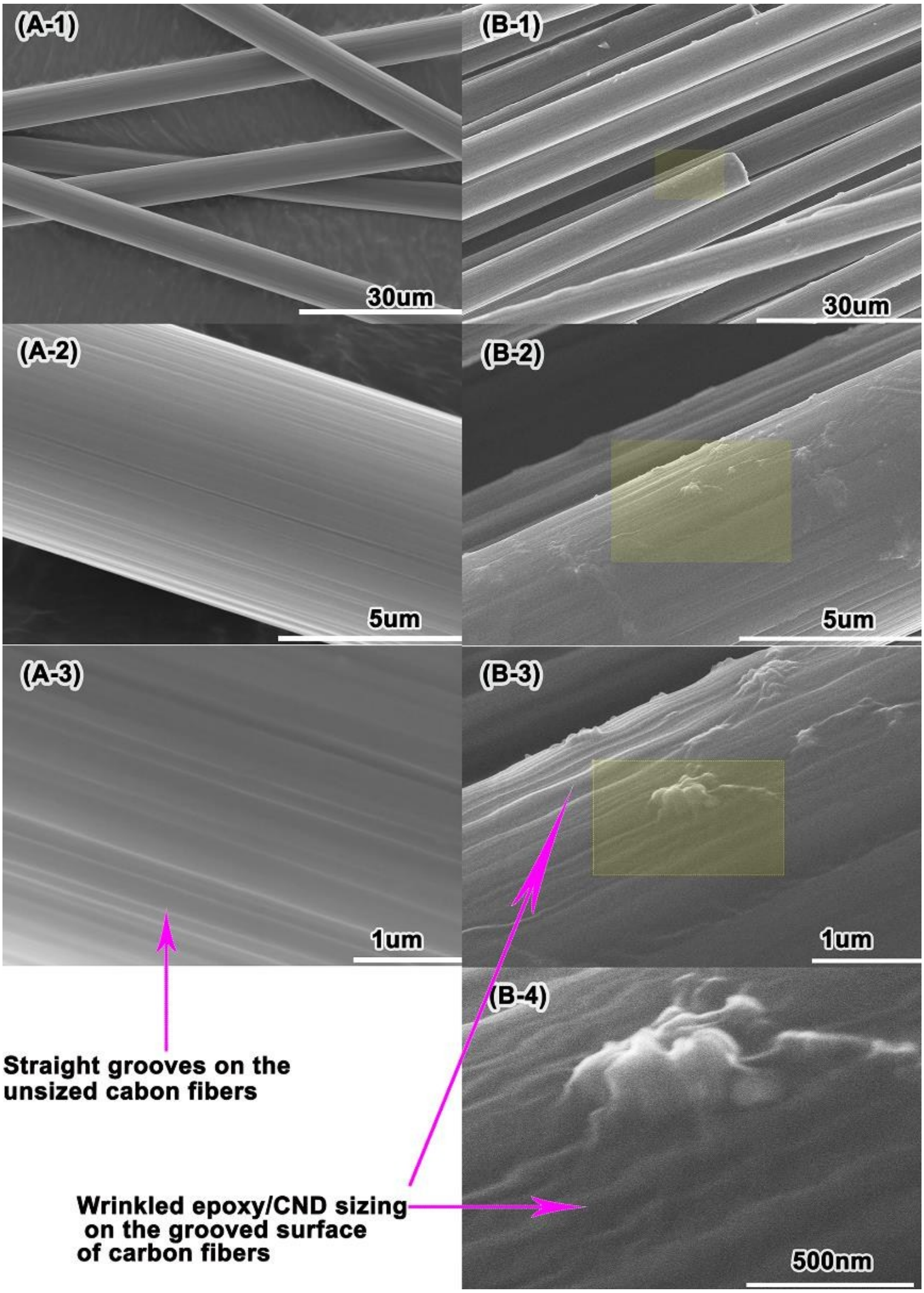

Figure S20. SEM images of the unsized CFs and epoxy/CND sized CFs. A1-3 are the unsized CFs. B1-4 are the epoxy/CND sized CFs.

21. Raman spectra of the CF samples

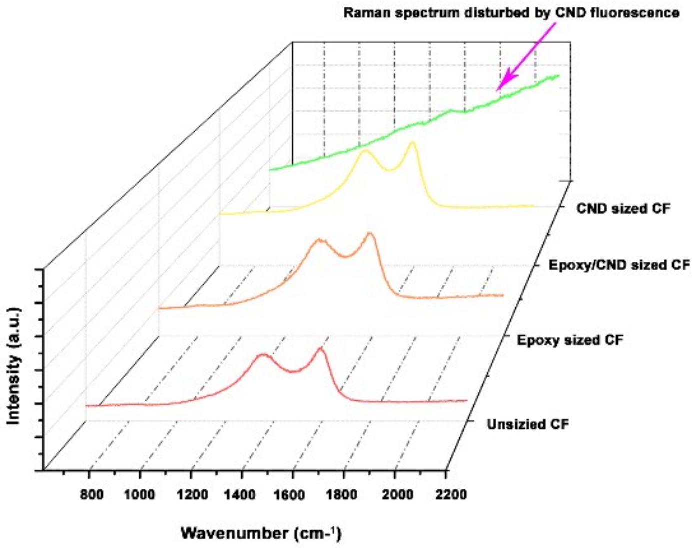

Figure S21. Raman spectra of the CF samples. CFs were tested without pre hot treatment.

22. SEM images of the CFs and CF/Epoxy composite rupture faces.

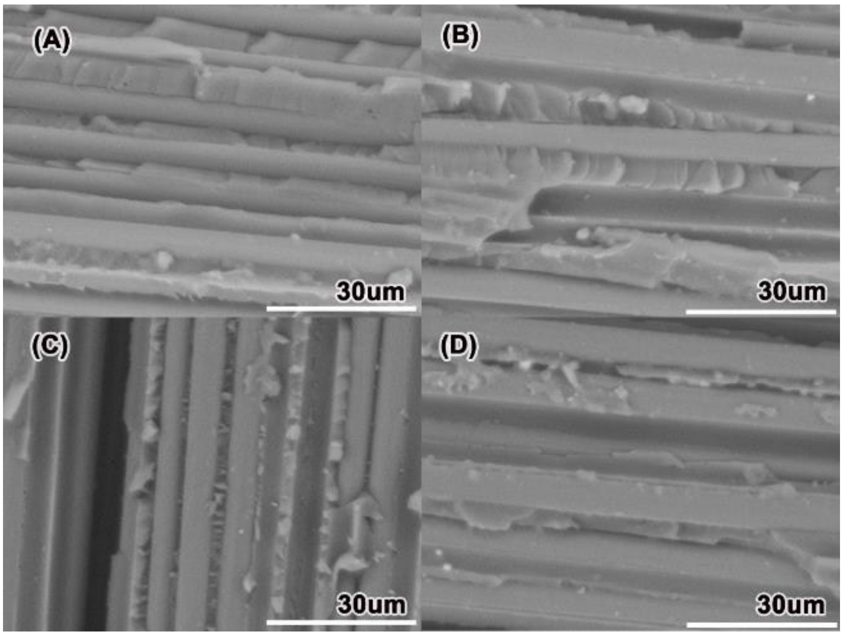

Figure S22. SEM images of the CF/epoxy composite rupture face. Unsized CF (A); epoxy sized CF (B); CND sized CF (C); epoxy/CND sized CF (D1).

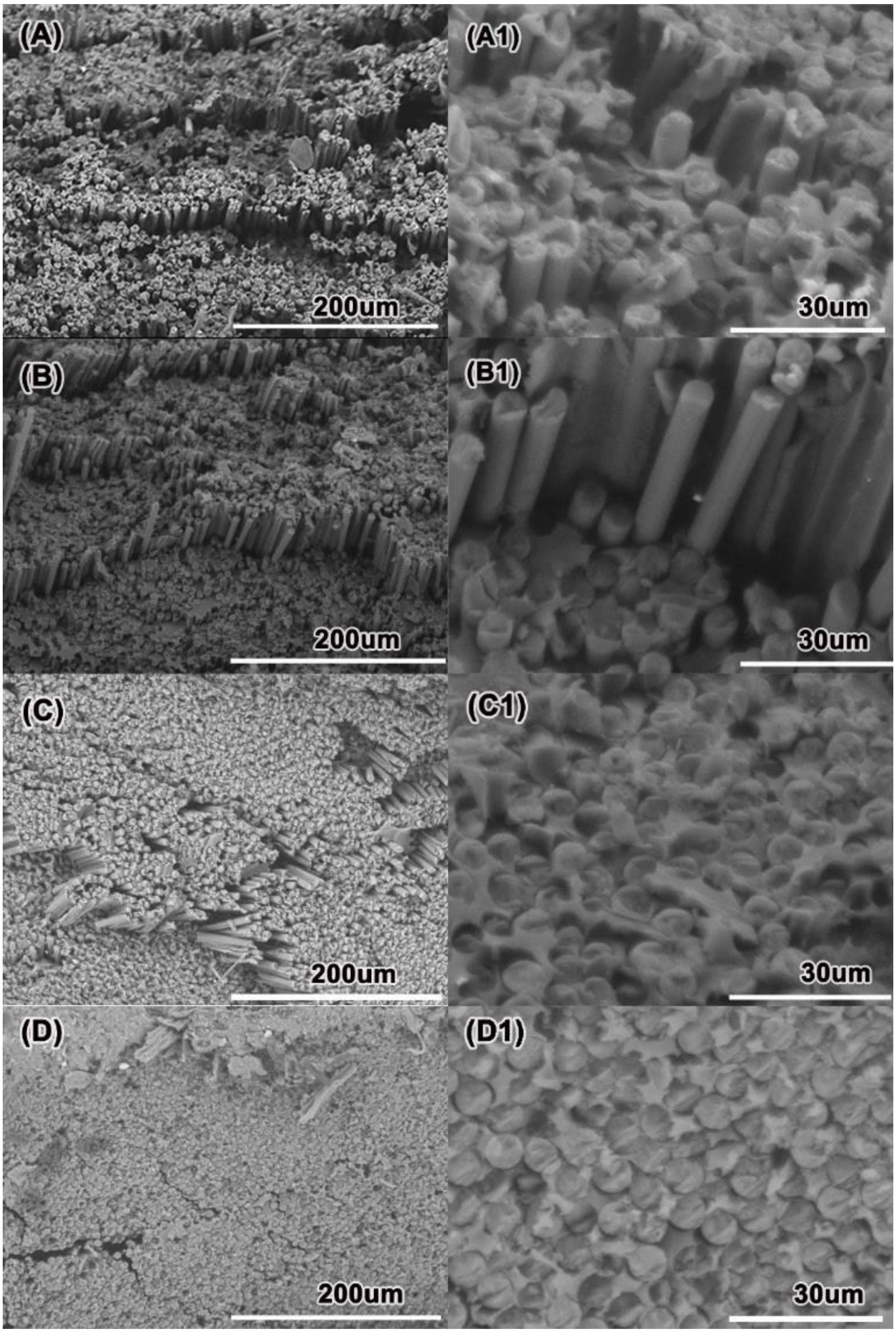

Figure S23. SEM images of the CF/epoxy composite rupture face. Unsized CF (A), (A1); epoxy sized CF (B), (B1); CND sized CF (C), (C1); epoxy/CND sized CF (D), (D1).

23. References in ESI

(1) Falco, C.; Baccile, N.; Titirici, M. M. Morphological and structural differences between glucose, cellulose and lignocellulosic biomass derived hydrothermal carbons. *Green Chem.* **2011**, *13*, 3273.

(2) Falco, C.; Caballero, F. P.; Babonneau, F.; Gervais, C.; Laurent, G.; Titirici, M. M.; Baccile, N. Hydrothermal carbon from biomass: structural differences between hydrothermal and pyrolyzed carbons via ^{13}C solid state NMR. *Langmuir* **2011**, *27*, 14460-14471.

(3) Li, W.; Wang, S.; Li, Y.; Ma, C.; Huang, Z.; Wang, C.; Li, J.; Chen, Z.; Liu, S. One-step hydrothermal synthesis of fluorescent nanocrystalline cellulose/carbon dot hydrogels. *Carbohydr. Polym.* **2017**, *175*, 7-17.

(4) Guiotoku, M.; Rambo, C.R.; Maia, C.M.B.F.; Hotza, D. Synthesis of carbon-based materials by microwave-assisted hydrothermal process. In: Chandra, U. editor. *Microwave heating*. New York: Intech; **2011**. p. *291–308*.

(5) Cai, W.; Piner, R. D.; Stadermann, F. J.; Park, S.; Shaibat, M. A.; Ishii, Y.; Yang, D.; Velamakanni, A.; An, S. J.; Stoller, M.; An, J.; Chen, D.; Ruoff, R. S. Synthesis and Solid-State NMR Structural Characterization of 13C-Labeled Graphite Oxide. *Science*, **2008**, *321*, 1815-1817

(6) Erdenetsogt, B. -O.; Lee, I.; Lee, S. K.; Ko, Y. -J.; Bat-Erdene, D., Solid-state ^{13}C CP/MAS NMR study of Baganuur coal, Mongolia: Oxygen-loss during coalification from lignite to subbituminous rank. *Int. J. Coal Geol.* **2010,** *82* (1-2), 37-44.

(7) Pan, N.; Guan, D.; Yang, Y.; Huang, Z.; Wang, R.; Jin, Y.; Xia, C., A rapid low-temperature synthetic method leading to large-scale carboxyl graphene. *Chem. Eng. J*, **2014**, *236*, 471-479.

(8) Ye, R.; Xiang, C.; Lin, J.; Peng, Z.; Huang, K.; Yan, Z.; Cook, N. P.; Samuel, E. L.; Hwang, C. C.; Ruan, G.; Ceriotti, G.; Raji, A. R.; Marti, A. A.; Tour, J. M., Coal as an abundant source of graphene quantum dots. *Nat. commun.* **2013**, *4*, 2943.

(9) Emiru, T.F.; Ayele, D.W., Controlled synthesis, characterization and reduction of graphene oxide: A convenient method for large scale production. *Egypt. J. Basic Appl. Sci.*, **2017,** *4*, 74-79

(10) He, D.; Peng, Z; Gong, W.; Luo, Y.; Zhao, P.; Kong, L., Mechanism of a Green Graphene Oxide Reduction with Reusable Potassium Carbonate. RSC Adv., **2015**, *5,* 11966-11972

(11) Liu, H.; Kuila, T.; Kim, N.H.; Ku, B.C; Lee, J.H., In situ synthesis of reduced graphene oxide/polyethyleneimine composite and its gas barrier properties. *J. Mater. Chem. A,* **2013**, *1*, 3739-3746

(12) Feng, L.; Zhao, G.; Zhao, Y.; Zhao, M.; Tang, J., Construction of the molecular structure model of the Shengli lignite using TG-GC/MS and FTIR spectrometry data. *Fuel* **2017,** *203*, 924-931.

(13) Lu, Y.; Feng, L.; Jiang, X.; Zhao, Y.; Zhao, G.; Yuan, C., Construction of a molecular structure model of mild-oxidized Chinese lignite using Gaussian09 based on data from FTIR, solid state ^{13}C-NMR. *J. Mol. Model.* **2018,** *24* (6), 135.

(14) Solum, M. S.; Pugmire, R. J.; Grant, D. M. ^{13}C Solid-state NMR of Argonne Premium Coals. *Energy Fuels* **1989**, *3*, 1987-1993.

(15) Solum, M. S.; Sarofim, A. F.; Pugmire, R. J.; Fletcher, T. H. and Zhang H. ^{13}C NMR Analysis of Soot Produced from Model Compounds and a Coal. *Energy Fuels* **2001**, *15*, 961-971.

(16) Liu, P.; Wang, L.; Zhou, Y.; Pan, T.; Lu, X.; Zhang, D. Effect of hydrothermal treatment on the structure and pyrolysis product distribution of Xiaolongtan lignite. *Fuel* **2016**, *164*, 110-118.

(17) Baysal, M.; Yürüm, A.; Yıldız, B.; Yürüm, Y., Structure of some western Anatolia coals investigated by FTIR, Raman, ^{13}C solid state NMR spectroscopy and X-ray diffraction. International. *Int. J. Coal Geol.* **2016**, *163*, 166-176.

(18) Cao, X.; Pignatello, J. J.; Li, Y.; Lattao, C.; Chappell, M. A.; Chen, N.; Miller, L. F.; Mao, J. Characterization of Wood Chars Produced at Different Temperatures Using Advanced Solid-State ^{13}C NMR Spectroscopic Techniques. *Energy Fuels* **2012**, *26*, 5983-5991.

(19) Wang, Z.; Opembe, N.; Kobayashi, T.; Nelson, N. C.; Slowing, I. I.; Pruski, M. Quantitative atomic-scale structure characterization of ordered mesoporous carbon materials by solid state NMR. *Carbon* **2018**, *131*, 102-110.

(20) Ben, H.; Ragauskas, A. J. NMR Characterization of Pyrolysis Oils from Kraft Lignin. *Energy Fuels* **2011**, 25, 2322-2332.

(21) Wang, Q.; Hou, Y.; Wu, W.; Yu, Z.; Ren, S.; Liu, Q.; Liu, Z., A study on the structure of Yilan oil shale kerogen based on its alkali-oxygen oxidation yields of benzene carboxylic acids, ^{13}C NMR and XPS. *Fuel Process. Technol.* **2017**, *166*, 30-40;

(22) Tong, J.; Han, X.; Wang, S.; Jiang, X. Evaluation of Structural Characteristics of Huadian Oil Shale Kerogen Using Direct Techniques (Solid-State ^{13}C NMR, XPS, FT-IR, and XRD). *Energy Fuels* **2011**, *25*, 4006-4013.

(23) Roberts, M. J.; Everson, R. C.; Neomagus, H. W. J. P.; Niekerk, V.D.; Mathews, J. P.; Branken, D. J. Influence of maceral composition on the structure, properties and behavior of chars derived from South African coals. *Fuel* **2015**, *142*, 9-20.

(24) Brech, Y. L.; Delmotte, L.; Raya, J.; Brosse, N.; Gadiou, R.; Dufour, A. High resolution solid state 2D NMR analysis of biomass and biochar. *Anal. Chem.* **2015**, *87*, 843-7.
